# Molecular ontology of the parabrachial nucleus

**DOI:** 10.1002/cne.25307

**Published:** 2022-02-21

**Authors:** Samyukta Karthik, Dake Huang, Yaritza Delgado, Justin J. Laing, Lila Peltekian, Gabrielle N. Iverson, Fillan Grady, Rebecca L. Miller, Corey M. McCann, Bernd Fritzsch, Igor Y. Iskusnykh, Victor V. Chizhikov, Joel C. Geerling

**Affiliations:** ^1^ Department of Neurology University of Iowa Iowa City Iowa USA; ^2^ Department of Anatomy and Neurobiology Washington University School of Medicine Saint Louis Missouri USA; ^3^ Iowa Neuroscience Institute Iowa City Iowa USA; ^4^ Department of Biology University of Iowa Iowa City Iowa USA; ^5^ Department of Anatomy and Neurobiology University of Tennessee Health Science Center Memphis Tennessee USA

**Keywords:** breathing, development, interoception, interoceptive, Kölliker–Fuse, pain, parabrachial complex, parabrachialis, pontine pneumotaxic center, pontine taste area

## Abstract

Diverse neurons in the parabrachial nucleus (PB) communicate with widespread brain regions. Despite evidence linking them to a variety of homeostatic functions, it remains difficult to determine which PB neurons influence which functions because their subpopulations intermingle extensively. An improved framework for identifying these intermingled subpopulations would help advance our understanding of neural circuit functions linked to this region. Here, we present the foundation of a developmental‐genetic ontology that classifies PB neurons based on their intrinsic, molecular features. By combining transcription factor labeling with Cre fate‐mapping, we find that the PB is a blend of two, developmentally distinct macropopulations of glutamatergic neurons. Neurons in the first macropopulation express *Lmx1b* (and, to a lesser extent, *Lmx1a*) and are mutually exclusive with those in a second macropopulation, which derive from precursors expressing *Atoh1*. This second, *Atoh1*‐derived macropopulation includes many *Foxp2*‐expressing neurons, but *Foxp2* also identifies a subset of *Lmx1b*‐expressing neurons in the Kölliker–Fuse nucleus (KF) and a population of GABAergic neurons ventrolateral to the PB (“caudal KF”). Immediately ventral to the PB, *Phox2b*‐expressing glutamatergic neurons (some coexpressing *Lmx1b*) occupy the KF, supratrigeminal nucleus, and reticular formation. We show that this molecular framework organizes subsidiary patterns of adult gene expression (including *Satb2*, *Calca*, *Grp*, and *Pdyn*) and predicts output projections to the amygdala (*Lmx1b*), hypothalamus (*Atoh1*), and hindbrain (*Phox2b*/*Lmx1b*). Using this molecular ontology to organize, interpret, and communicate PB‐related information could accelerate the translation of experimental findings from animal models to human patients.

## Graphical abstract

The parabrachial nucleus (PB) contains diverse, intermingled neurons regulating various homeostatic functions. Their neuronal distributions often do not align with cytoarchitectural subdivisions. Here, we show that the PB is a blend of two, developmentally distinct glutamatergic populations identified by the embryonic transcription factors *Atoh1* and *Lmx1a*/*Lmx1b*. We show that macropopulation identity predicts output connectivity and other gene expression patterns in this region. Our findings lay the foundation for a molecular ontology that will be useful for interpreting transcriptomic, connectomic, and other experimental data involving PB neurons.

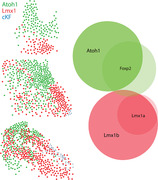

## 1 INTRODUCTION

A complex region of the brainstem tegmentum links the rhombencephalon (pons‐medulla) and mesencephalon (midbrain). Embryonic precursors here generate a variety of neurons in the brainstem and the entire cerebellum (Ben‐Arie et al., 1997; Chizhikov et al., 2006; Hoshino et al., 2005; Liu et al., 2010; Machold & Fishell, 2005; Rose et al., 2009; Wang et al., 2005; Watson et al., 2017; Yamada et al., 2007). In the dorsolateral corner of this region, a diverse constellation of neurons communicates with every major region of the central nervous system, from cerebral cortex to spinal cord (Herbert et al., 1990; Huang et al., 2020; Moga et al., 1990; Saper & Loewy, 1980). Herrick first identified this region as the “superior secondary gustatory nucleus” in fish (1905), and subsequent work identified taste‐relay neurons in rats (Norgren & Leonard, 1971). These neurons surround the *brachium conjunctivum* (superior cerebellar peduncle) and are referred to as the “parabrachial” nucleus (PB).

Over the past 50 years, experiments in rats and mice have implicated the PB in many more homeostatic functions, including hunger, thirst, sodium appetite, taste aversion, and cardiorespiratory control (Chamberlin & Saper, 1994; Geerling & Loewy, 2007; Kim et al., 2020; Menani et al., 2014; Palmiter, 2018; Ryan et al., 2017). It is also clear that the PB is a lynchpin in the neurobiological basis of pain, itch, and thermoregulation (Barik et al., 2021; Bernard & Besson, 1990; Bernard et al., 1994; Bester et al., 1995; Bourgeais et al., 2001; Cechetto et al., 1985; Chiang et al., 2019; Chiang et al., 2020; Coizet et al., 2010; Deng et al., 2020; Gauriau & Bernard, 2002; Morrison & Nakamura, 2019; Mu et al., 2017; Palmiter, 2018). Some of these functions were linked to specific subsets of PB neurons, but the inscrutability of remaining subpopulations limits our understanding of several important brain circuits.

One reason these neurons remain enigmatic is that, rather than forming discrete layers or nuclei (as in the cerebral cortex or thalamus), most PB subpopulations form an intermingled, three‐dimensional web. The current method of classifying PB neurons uses the superior cerebellar peduncle to split the region into a medial and a lateral subdivision (Taber, 1961). Next, subnuclear boundaries are applied within each subdivision, using cytoarchitectural criteria proposed in rats (Fulwiler & Saper, 1984). While this cytoarchitectural taxonomy facilitated initial progress, adapting these criteria between species proved challenging. Even between rats and mice, species differences led to confusion regarding the location and identity of several subpopulations. Prominent examples include rostral PB neurons that relay thermosensory information to the hypothalamus (Geerling et al., 2016; Nakamura & Morrison, 2008, 2010), dorsal PB neurons that relay pain‐related information to the thalamus (Barik et al., 2021; Bourgeais et al., 2001), and caudal PB neurons that influence appetite (Gasparini et al., 2021; Gasparini et al., 2019; Geerling & Loewy, 2007; Geerling et al., 2011; Gong et al., 2020; Jarvie & Palmiter, 2017; Kim et al., 2020; Li et al., 2019; Park et al., 2020; Resch et al., 2017). Our efforts to adapt this taxonomy from rats to mice made it clear that some cytoarchitectural criteria simply do not translate (Gasparini et al., 2021; Gasparini et al., 2019; Geerling et al., 2016), and previous attempts to describe and organize this region suggest further differences in the human brainstem (Block & Estes, 1990; de Lacalle & Saper, 2000; Gioia et al., 2000; Lavezzi et al., 2004; Ohm & Braak, 1988; Pammer et al., 1988; Petrovicky, 1989; Rub et al., 2001).

In addition to species differences, an unavoidable challenge when applying cytoarchitectural criteria is that this requires interpreting Nissl‐stained tissue, an inherently subjective activity (Swanson, 2000). Distinctions can be subtle, and even experts cannot distinguish functionally diverse neurons if they have the same Nissl‐stained appearance. A more ideal framework for classifying diverse, intermingled neurons would incorporate observer‐independent, molecular information as a core feature. Using gene expression to classify neurons can produce results resembling cytoarchitectural analysis, while also distinguishing new subpopulations (Ortiz et al., 2020). Gene expression predicts the output connectivity of PB neurons (Huang et al., 2020; Huang et al., 2021), and basing a neuronal ontology on molecular and connectomic features could offer a more accessible, universal language for interpreting and communicating experimental findings (Bota & Swanson, 2008; Hamilton et al., 2012; Larson & Martone, 2009; Zeng & Sanes, 2017).

In the PB, molecular information has not kept pace with other brain regions. Limited information suggests that PB neurons derive from precursors in the embryonic isthmus (r0; Watson et al., 2017) and rhombomere 1 (r1; Figure 2M of Machold & Fishell, 2005). At these levels of the neural tube, PB neurons are thought to arise from precursors in the rhombic lip that express *Atoh1* (Machold & Fishell, 2005; Rose et al., 2009; van der Heijden & Zoghbi, 2018; Wang et al., 2005), but we lack information on the relationship between *Atoh1*‐derived neurons and adult subpopulations expressing a potpourri of transcription factors (including *Foxp2*, *Lmx1b*, *Lmx1a*, *Satb2*, and *Runx1*), neuropeptides, receptors, and other genetic markers in current use (Asbreuk et al., 2002; Chizhikov et al., 2021; Dai et al., 2008; Gray, 2008; Guo et al., 2007; Hernandez‐Miranda et al., 2017; Kang et al., 2007; Liu et al., 2010; Maeda et al., 2009; Miller et al., 2012; Millonig et al., 2000; Mishima et al., 2009; Zagami & Stifani, 2010; Zou et al., 2009). These markers identify an incomplete patchwork of PB neurons, and our ability to study neural circuit functions involving the remaining subpopulations would benefit from a more comprehensive framework.

As a first step, we sought a framework of developmental‐genetic information that identifies all PB neurons. Motivated by novel activity and connectivity patterns in rats (Geerling & Loewy, 2006b, 2007; Geerling et al., 2010), this project began with the observation that two transcription factors have complimentary distributions in this region (Geerling et al., 2011; Miller et al., 2012; Shin et al., 2011). Here, we combine Cre fate‐mapping, mRNA labeling, immunolabeling, and axonal tracing to replicate and extend these observations in mice.

Our new findings reveal that “the” PB does not have a single developmental origin. Its glutamatergic neurons are a blend of two, mutually exclusive macropopulations, defined by the embryonic transcription factors *Lmx1b* and *Atoh1*. We show that this framework clarifies the identity and connectivity of further PB subpopulations and challenges previous ideas about the composition and origin of the Kölliker–Fuse nucleus (KF). Together, these findings lay a developmental‐genetic foundation for a molecular ontology that investigators can use to identify and target PB neurons.

## 2 MATERIALS AND METHODS

### 2.1 Animals

We used a total of *n* = 68 male and female mice, aged 7–23 weeks and weighing 17–31 g. All mice were group‐housed in a temperature‐ and humidity‐controlled room on a 12/12‐h light/dark cycle and had ad libitum access to standard rodent chow and water. In addition to C57B6/J mice (Jackson Laboratories), we used a variety of knockin‐Cre and Cre‐reporter mice. Detailed information about each strain is provided in Table 1. For all mRNA and protein labeling that did not require a Cre‐reporter, we replicated labeling in at least three, 8−12‐week‐old C57B6/J mice. All mRNA and protein labeling in Cre‐reporter mice was replicated in at least three mice with a hemizygous Cre allele and a hemizygous Cre‐reporter allele. Stereotaxic injections, histologic procedures, and confocal microscopy in *n* = 4 Harlan Sprague–Dawley rats were performed as described previously (Geerling & Loewy, 2007; Geerling et al., 2011; Shin et al., 2011) and in accordance with the guidelines of the Institutional Animal Care and Use Committee at Washington University in Saint Louis. All experiments in mice were conducted in accordance with the guidelines of the Institutional Animal Care and Use Committee and at the University of Iowa.

**TABLE 1:** Cre‐driver and ‐reporter mice

Strain	References	Source information	Key Gene
*Atoh1‐Cre*	Matei et al. *Dev Dyn* 234(3):633‐50. 2005	Bernd Fritzsch, University of Iowa Jax 011104 https://www.jax.org/strain/011104	Transgenic construct containing the JQ2‐*Atoh1* promoter fragment ligated to the bacteriophage P1 Cre recombinase
*Lmx1a‐Cre*	Victor V. Chizhikov, Anne G. Lindgren, D. Spencer Currle, Matthew F. Rose, Edwin S. Monuki, Kathleen J. Millen Development 2006 133: 2793–2804; doi: 10.1242/dev.02441	Victor V. Chizhikov, University of Tennessee HSC http://www.informatics.jax.org/allele/key/569853	BAC transgenic construct containing ∼200 kb of the mouse *Lmx1a* locus with *Lmx1a* coding sequence replaced by *Cre* coding sequence
*Vglut2‐IRES‐Cre* *(Slc17a6‐IRES‐Cre)*	Vong, Linh, et al. “Leptin action on GABAergic neurons prevents obesity and reduces inhibitory tone to POMC neurons.” *Neuron* 71.1 (2011): 142–154	Jax 016963 https://www.jax.org/strain/016963	IRES‐Cre inserted downstream of the stop codon of *Slc17a6* on chromosome 7
*Vgat‐IRES‐Cre* (*Slc32a1‐IRES‐Cre*)	Vong L, Ye C, Yang Z, Choi B, Chua S, Lowell BB. Leptin action on GABAergic neurons prevents obesity and reduces inhibitory tone to POMC neurons. *Neuron*. 2011; 71:142−154	Jax 028892 https://www.jax.org/strain/028862	IRES‐Cre inserted after the *Slc32a1* stop codon
Ai9(*R26‐lsl‐ tdTomato*)	Madisen L; Zwingman TA; Sunkin SM; Oh SW; Zariwala HA; Gu H; Ng LL; Palmiter RD; Hawrylycz MJ; Jones AR; Lein ES; Zeng H. 2010. A robust and high‐throughput Cre reporting and characterization system for the whole mouse brain. *Nat Neurosci* 13(1):133‐40	Jax 007909 https://www.jax.org/strain/007909	tdTomato (red fluorescent protein) insertion after lox‐STOP‐lox at Rosa26 locus
Ai14(*R26‐lsl‐tdTomato*) reporter	Madisen L; Zwingman TA; Sunkin SM; Oh SW; Zariwala HA; Gu H; Ng LL; Palmiter RD; Hawrylycz MJ; Jones AR; Lein ES; Zeng H. 2010. A robust and high‐throughput Cre reporting and characterization system for the whole mouse brain. *Nat Neurosci* 13(1):133‐40	Jax 007914 https://www.jax.org/strain/007914	Rosa‐CAG‐LSL‐tdTomato‐WPRE targeting vector inserted between exon 1 and 2 of the Gt(ROSA)26Sor locus
*R26‐LSL‐L10GFP* reporter	Krashes, Michael J., et al. “An excitatory paraventricular nucleus to AgRP neuron circuit that drives hunger.” *Nature* 507.7491 (2014): 238	Available from originating investigators http://www.informatics.jax.org/allele/MGI:5559562	Floxed transcription STOP cassette followed by EGFP/Rpl10 fusion reporter gene under control of the CAG promoter targeted to the Gt(ROSA)26Sor locus
*Pdyn‐IRES‐Cre*	Krashes, Michael J., et al. “An excitatory paraventricular nucleus to AgRP neuron circuit that drives hunger.” *Nature* 507.7491 (2014): 238	Jax 027958 https://www.jax.org/strain/027958	IRES‐Cre inserted downstream of the endogenous Pdyn (prodynorphin) gene
*Calca‐Cre (Calca‐tm1.1‐Cre‐EGFP)*	Carter, M., Soden, M., Zweifel, L. et al. “Genetic identification of a neural circuit that suppresses appetite.” *Nature* 503, 111−114 (2013)	Richard Palmiter, University of Washington (shared by Andrew Russo, University of Iowa)	Cre:GFP inserted downstream of the endogenous CGRP (calcitonin‐gene‐related‐peptide) gene

### 2.2 Stereotaxic injections

Mice were anesthetized with isoflurane and placed in a Kopf 1900 stereotactic frame. After midline incision, the skin was retracted to expose the skull and locate bregma. We injected the cholera toxin B‐subunit (CTb, 0.1% in distilled water; List, lot #10331A1) in *Atoh1‐Cre;R26‐lsl‐L10GFP* mice. We made nanoliter injections through a fine‐tipped micropipette (20–30 μm inner diameter) using controlled puffs of compressed air, typically 0.5–1 per second, with a target rate of 5–10 nl/min. Injection volumes ranged 15−45 nl. Stereotaxic targets included the central nucleus of the amygdala (1.0 mm caudal, 2.5 mm right, and 5.0−5.5 mm deep to bregma), insular cortex (0.0−0.5 mm caudal, 3.75−3.8 mm right, and 4.0−4.3 mm deep to bregma), and ventromedial hypothalamus (1.1−1.4 mm caudal, 0.06−0.55 mm right, and 5.6–5.9 mm deep to bregma). Each injection was made over a 5‐min period using picoliter air puffs through a solenoid valve (Clippard EV 24V DC) pulsed by a Grass stimulator. The pipette was left in place for an additional 3−5 min, then slowly withdrawn before the skin was closed using Vetbond (3 M). Carprofen (1 mg/kg s.c.) was provided for postoperative analgesia. We injected an additional series of C57B6/J mice with CTb, targeting the medullary reticular formation (6.3 mm caudal, 1.2 mm right, and 6.0 mm deep to bregma; 45 nl). Mice were kept alive for 3−5 days for retrograde axonal transport, and then perfused as described below. Synaptophysin‐mCherry labeling shown in the midbrain of *Pdyn‐IRES‐Cre* and *Calca‐Cre* mice represents unpublished tissue sections from two previous studies (for details, see Huang et al., 2020; Huang et al., 2021).

### 2.3 Perfusions and tissue sections

All mice were deeply anesthetized with ketamine‐xylazine (i.p. 150–15 mg/kg) and then perfused transcardially with phosphate‐buffered saline (PBS, prepared from 10x stock; P7059, Sigma), followed by 10% formalin‐PBS (SF100, Fisher Scientific). After perfusion, the brain was removed and fixed overnight in 10% formalin‐PBS at 4°C, then submerged in 30% sucrose‐PBS at 4°C for an additional day. Each brain was sectioned into 40 μm‐thick axial (coronal) slices using a freezing microtome. Three adjacent (1‐in‐3) tissue series were collected from each brain in separate tubes (labeled “A,” “B,” and “C”) containing a cryoprotectant solution of 35% (v/v in PBS) ethylene glycol (102466, Sigma‐Aldrich) and 25% glycerol (G22025, RPI). These tubes were stored at −20°C until further processing. Each brain, therefore, yielded three sets of axial tissue sections that allowed us to study up to three separate combinations of molecular markers in the PB region from each mouse. For experimental replication of each endpoint, we analyzed brain tissue from at least three separate mice.

### 2.4 Immunofluorescence

We removed tissue sections from cryoprotectant and selected sections of interest (typically, a series of nine tissue sections containing the full PB region or CTb injection site; otherwise, a full‐brain series). After rinsing the sections in PBS, we incubated them with primary antisera (Table 2) in a PBT‐NDS‐azide solution comprised of PBS with 0.25% Triton X‐100 (BP151‐500, Fisher), 2% normal donkey serum (NDS, 017‐000‐121; Jackson ImmunoResearch), and 0.05% sodium azide (14314, Alfa Aesar) as a preservative. The sections were incubated in this primary antibody solution overnight at room temperature on a tissue shaker. The next morning, sections were washed three times in PBS, then incubated for 2 h at room temperature with species‐specific secondary antibodies conjugated to Cy3, Cy5, Alexa Fluor 488, or biotin (diluted 1:500−1000; Jackson ImmunoResearch) in PBT‐NDS‐azide. If a biotinylated secondary antibody was used, tissue sections were then washed three times and incubated for 2 h in streptavidin‐Cy5 (#SA1011, Invitrogen) or streptavidin‐Pacific Blue (#S11222, Invitrogen), diluted 1:1000 in PBT‐NDS‐azide. The sections were then washed three times in PBS, mounted on glass slides (#2575‐PLUS; Brain Research Laboratories), dried, and then coverslipped using Vectashield (Vector Laboratories) or Vectashield with DAPI (if no blue fluorophore was used). Slides were stored at 4°C until imaging.

**TABLE 2:** Antisera

Antigen	Immunogen description	Source, host species, RRID	Concentration
Choline acetyltransferase (ChAT)	Human placental choline acetyltransferase	Millipore, goat polyclonal, #AB144P, lot: JC1618187; RRID: AB_2079751	1:1000
Forkhead box protein 2 (FoxP2)	Recombinant human FOXP2 isoform 1 Ala640‐Glu715	R&D Systems, sheep polyclonal #AF5647; RRID: AB_2107133	1:10,000
LIM homeobox transcription factor 1 beta (Lmx1b) in rat	Three fusion proteins containing amino acid residues 17−47, 157−195, or 255−324 of mouse Lmx1b	Y. Ding, Chinese Academy of Science, Shanghai; rabbit polyclonal; RRID: AB_2314751 (no labeling in Lmx1b KO mice; Dai et al *J Comp Neurol* 2008)	1:1000
Lmx1b (mouse)	Full‐length LIM homeobox transcription factor 1 beta protein from mouse	C. Birchmeier, Max Delbruck Center for Molecular Medicine, Berlin; guinea pig polyclonal; RRID: AB_2314752	1:8000
mCherry	Full‐length mCherry fluorescent protein	Life Sciences, rat monoclonal, #M11217, lot: R1240561; RRID: AB_2536611 (we find no immunolabeling in mice without dsRed‐derived fluorescent proteins)	1:2000
Paired‐like homeobox 2b (Phox2b)	BSA‐coupled 15mer corresponding to the C terminus of the Phox2b protein with an added N‐terminal tyrosine	H. Enomoto, School of Medicine at Kobe University, Japan; RRID: AB_2895590	1:12,000
Paired‐like homeobox 2b (Phox2b)	Mouse monoclonal antibody raised against amino acids 11–70 mapping near the N‐terminus Phox2b of human origin	Santa Cruz, mouse monoclonal, #sc‐376997, lot: E1719; RRID: AB_2813765	1:1000
Special AT‐rich sequence‐binding protein 2 (Satb2)	Synthetic peptide within human SATB2 (proprietary sequence)	Abcam, rabbit monoclonal, cat. # ab92446, lot: GR325015−2, RRID: AB_1056367	1:3000
Tyrosine hydroxylase (TH)	Purified, SDS‐denatured rat pheochromocytoma TH	Millipore, mouse monoclonal, #MAB318, lot: NG1802536; RRID: AB_2201528	1:2000
Tyrosine hydroxylase (TH)	Denatured TH from rat pheochromocytoma (denatured by sodium dodecyl sulfate)	Millipore, rabbit polyclonal, #AB152, lot: 240602; RRID: AB_696697	1:10,000

### 2.5 In situ hybridization

We used RNAscope probes to label a variety of mRNA transcripts (see Table 3 for detailed information on each probe). For diaminobenzidine (DAB) labeling of single transcripts, we used the HD Reagent Kit (ref #322310; Advanced Cell Diagnostics). For fluorescence labeling of two or three transcripts in combination, we used the Fluorescent Multiplex Detection Reagents kit (ref# 320851; Advanced Cell Diagnostics).

**TABLE 3:** RNAscope probes

Probe	Common name	Channel	ACD catalog #	Lot #
Mm‐Calca‐C2	Calca	C2	417961‐C2	18165A
Mm‐FoxP2‐C3	FoxP2	C3	428791‐C3	17013A
MM‐Grp‐C2	Grp	C2	317861‐C2	19211A
Mm‐Lmx1a	Lmx1a	C1	493131	20042B
Mm‐Lmx1b	Lmx1b	C1	412931	18255B
Mm‐Pdyn‐C3	Pdyn	C3	318771‐C3	17290A
Mm‐Phox2b‐C2	Phox2b	C2	407861‐C2	19179B
Mm‐Slc6a5‐C1	GlyT2	C1	409741	16347A
Mm‐Slc17a6	Vglut2	C1	545891‐C1	17251A
Mm‐Slc32a1‐C2	Vgat	C2	319191‐C2	16340A
Mm‐Ubc‐C2	Ubc	C2	310771‐C2	18098B

For brightfield (DAB) labeling, we removed tissue from cryoprotectant, selected 6−8 sections per case containing the PB region, rinsed them in PBS, then incubated sections for 30 min in 0.3% hydrogen peroxide (#H325‐100, Fisher) to quench endogenous peroxidase activity. They were again washed in PBS, then mounted onto glass slides to dry overnight at room temperature. The following day, sections were dehydrated for 5 min in 100% EtOH, then dried at room temperature for 20 min. Next, sections were incubated in 1x Target Retrieval solution (ACD; 10 ml in 90 ml ddH2O, warmed for 5 min prior to slide incubation) for 5 min in a steamer (Oster, prewarmed for 60 min), then rinsed twice with ddH2O. Slides were next submerged in 100% EtOH for 1 min, then removed and dried at room temperature for 20 min. Next, we used an ImmEdge PAP pen (#H‐4000; Vector) to create a hydrophobic barrier around the sections, added four drops of Protease Plus (Ref 322331, Lot # 2000898; ACD), and placed slides in a covered glass petri dish floating in a 40°C water bath for 30 min. After washing twice with ddH2O, we covered sections with a C1 probe (prewarmed to 40°C for 10 min) for 2 h at 40°C. Next, the slides were washed twice with 1X RNAscope Wash Buffer, then incubated with four drops of AMP 1 (Ref 322311, Lot# 2008215, ACD) for 30 min at 40°C. This process of washing twice with 1X Wash Buffer and then incubating was repeated for AMPs 2−6 with the following times and temperatures: AMP 2 (Ref 322312, Lot# 2007617, ACD), 15 min at 40°C; AMP 3 (Ref 322313, Lot# 2008317), 30 min at 40°C; AMP 4 (Ref 322314, Lot# 2007619), 15 min at 40°C; AMP 5 (Ref 322315, Lot # 2007620), 30 min at room temperature; and AMP 6 (ref 322316, Lot# 2007621) room temperature for 15 min. After two final washes in 1X Wash Buffer, we mixed equal volumes of “DAB‐A” and “DAB‐B” (ACD), then added this combined solution to each slide for 10 min. Finally, we dipped slides in ddH2O, then dehydrated the sections in a series of EtOH solutions (50%, 75%, and 100%) for 2 min each followed by two xylene solutions for 5 min each. After removal from xylenes, the slides were coverslipped immediately with Cytoseal (#8310‐16 Thermo Scientific) and stored at room temperature.

For fluorescence labeling, we removed tissue from cryoprotectant, selected 6−8 sections containing the PB region, rinsed them in PBS, and mounted them on glass slides to dry overnight. In the morning, after dehydrating sections in an ascending series of alcohols (50%, 70%, 100%, and 100% EtOH; 5 min each), we used an ImmEdge PAP pen to create a hydrophobic barrier around the sections, then washed in PBS twice, for 2 min each, at room temperature. The sections were then covered with Protease IV and placed in a covered glass petri dish, floating in a 40°C water bath, for 30 min. After washing twice in PBS, the sections were incubated in a combination of two or three probes for 2 h at 40°C. After that, AMPs 1−4 were added, in series, for 15−30 min each, at 40°C, with two, 2‐min rinses in 1X RNAscope Wash Buffer (#320058; diluted 1:50 in ddH20) between each step. After a final wash in PBS, the slides were dried at room temperature and coverslipped using Vectashield with DAPI.

### 2.6 Imaging, analysis, and figure preparation

We used an Olympus VS120 slide‐scanning microscope for brightfield and epifluorescence imaging, collecting whole‐slide images of all tissue in this study. For brightfield imaging, after acquiring a 2× overview scan of the full slide, we used a 10× (NA 0.40) objective to image a single, central focal plane through all sections, then a 20× (NA 0.75) objective to collect an 11‐focal‐plane “extended focal image” (EFI) through each section. For epifluorescence imaging, after a 2× overview scan, we acquired all color channels in a single, central focal plane using the same 10× objective, then used the same 20× objective to collect EFI images of the midbrain‐hindbrain tegmentum (including the full, bilateral PB and neighboring structures, at all rostrocaudal levels). In some cases, we also collected multifocal Z‐stacks with a 40× (NA 0.95) air objective in smaller regions of interest. For each slide, this produced a Virtual Slide Image file containing a 2× overview layer (covering the whole slide), plus a 10× layer (containing all tissue sections) and additional layers containing 20× EFI and, in some cases, 40× virtual Z‐stacks in regions of interest.

After reviewing all images in VS‐ASW (Olympus) or OlyVIA (Olympus), we used cellSens (Olympus) to assign a color to each grayscale acquisition channel and to export cropped, full‐resolution images. We used Adobe Photoshop to adjust brightness and contrast. For each analysis, we selected 6−8 evenly spaced, axial tissue sections from *n* = 3 cases with high‐quality histology across all rostral‐to‐caudal levels of the PB.

In Adobe Illustrator, we placed a symbol atop each cell nucleus containing immunofluorescence labeling for Lmx1b, Foxp2, or Phox2b; cytoplasmic GFP (Cre‐reporter for *Atoh1‐Cre*); or nuclear and cytoplasmic tdTomato (Cre‐reporter for *Lmx1a‐Cre*). We labeled cells containing each marker using a separate symbol, in a separate Illustrator layer, and used additional symbols (in additional, separate layers) for all combinations of double‐ and triple‐labeling. We marked only cells that contained in‐focus labeling. All counting layers were reviewed, in every section, by a senior neuroanatomist (J.C.G.), and both S.K. and J.C.G. reviewed all plots and source images at high magnification to reach consensus. We only included labeling with fluorescence intensity that both investigators agreed to be above the background fluorescence of surrounding tissue and different from any lipofuscin autofluorescence that appeared (to a varying extent across cases) as small, punctate clusters of intracellular debris distinguished by identical fluorescence emission in both the red and green channels. Lipofuscin autofluorescence in this region is most prominent in the mesencephalic trigeminal nucleus and becomes more prominent widespread in older adults, so we focused on young adult mice (8–12 weeks).

As medial and lateral boundaries of our PB counting region, we used the lateral surface of Barrington's nucleus and the lateral brainstem surface. At caudal levels, our dorsal boundary was the dorsal brainstem surface and fourth ventricular ependyma. At middle to rostral levels (“mesencephalic” PB), our dorsal boundaries were the ventral edge of the cuneiform nucleus and the ventrolateral surface of the periaqueductal gray matter. Our ventral and caudal boundaries were the dorsal surfaces of the vestibular nuclear complex and of the principal sensory (PSV) and motor (V) trigeminal nuclei. We did not count cells beyond the ventral or rostral limits of the KF and supratrigeminal nuclei, nor did we count neurons in the pedunculopontine tegmental nucleus or in the dorsal or ventral nuclei of the lateral lemniscus.

We counted the symbols in each layer by first locking other layers, then using “Document Info > Objects” in Illustrator, and then entered each count into a Microsoft Excel spreadsheet for further analysis. We also used Illustrator to make drawings, arrange images, and add lettering for figure layouts. Scale bars were traced in Illustrator atop calibrated lines from cellSens to produce clean white or black lines in each figure.

We applied the Abercrombie correction factor to compensate for overcounting (Guillery, 2002). This correction factor requires knowing the section thickness and the diameter of the key counting element, which was the cell nucleus in our analyses. We, therefore, measured the diameter of FoxP2‐, Lmx1b‐, and Phox2b‐immunoreactive nuclei, as well as the nuclear‐void diameter in L10GFP‐expressing neurons (in *Atoh1‐Cre;R26‐lsl‐L10GFP* mice). For each cell type, 30 nuclei were selected at random and measured from each case (∼3 per marker, per section), across all *Atoh1‐Cre;R26‐lsl‐L10GFP* cases, every *Lmx1a‐Cre;R26‐lsl‐tdTomato* case, and all C57B6/J cases that were analyzed for Phox2b, Lmx1b, and/or FoxP2 counts. Average total nuclear diameters in all groups ranged 9.0−9.5 μm. As a rough estimate of the total number of neurons in the mouse PB, we added together the individual counts of each cell population, then subtracted the numbers of double‐ and triple‐labeled neurons. Finally, since we worked with tissue subsamples (1‐in‐3 tissue series), we multiplied the overall, Abercrombie‐corrected average sum of PB neurons by 3 to estimate the total number.

In this article, we use the term “mutual exclusivity” to mean that markers are found in largely separate populations that are at least 98−99% distinct from one another. Most histologic techniques for labeling protein or mRNA expression do not produce 100% mutually exclusive labeling, and a lack of perfect mutual exclusivity between a pair of mRNA transcripts, immunolabeled proteins, or fluorescent protein reporters may involve background thresholding as much as actual coexpression. In this study, we did not include faint or ambiguous labeling within the range of background tissue fluorescence. We analyzed most cells and cell populations in relatively thick (40 μm) tissue sections at digital magnifications spanning ∼10−100 μm (not submicron or subcellular distributions), which is optimal for comprehensively analyzing macropopulation‐level features throughout the full, three‐dimensional extent of the PB complex, which encompasses ∼1−2 mm^3^. Wherever we found clear‐cut examples of colocalized markers, we describe our observations in detail.

### 2.7 Nomenclature

For rat and mouse genes, we used MGI nomenclature. For rat and mouse proteins and Cre‐reporters, we used common abbreviations from the published literature. For neuroanatomical structures and cell populations, where possible, we use and refer to nomenclature defined in peer‐reviewed neuroanatomical literature. In some instances, we use or refer to nomenclature derived from rodent brain atlases (Dong, 2008; Paxinos & Franklin, 2013; Paxinos & Watson, 2007; Swanson, 1992). For the PB region specifically, mutual inconsistencies among existing systems of nomenclature (both between atlases and within the current literature) is a core topic addressed by the experimental results and analysis in this study.

Initially, we did not plan to focus attention on the region ventrolateral to the rostral PB that is referred to as the “Kölliker–Fuse nucleus,” but neurons in this location appeared as an emergent property in several of our results. These eponyms are historically inaccurate, and both the location and neuronal composition of the KF are defined inconsistently (Petrovicky, 1989), but we use the term “KF” to indicate a small region that is ventral to the rostral, ventrolateral tip of the PB at levels approximately 4.8−5.0 mm caudal to bregma (Paxinos & Franklin, 2013). This location borders a small white matter tract, labeled “ventral spinocerebellar tract” in rodent brain atlases (Dong, 2008; Paxinos & Franklin, 2013), which extends past the cerebellum rostrally, alongside the lateral PB, and then ventrally, alongside the KF. As described below, novel molecular features in this location identify and distinguish intermixed populations of neurons. The objective of this study is to maximize reproducibility by focusing on observer‐independent, molecular properties that unambiguously identify populations of neurons, rather than boundary lines that are inferred from Nissl cytoarchitecture. Consequently, and by design, no results or conclusions in this study were based on or inferred from Nissl‐stained tissue.

## 3 RESULTS

### 3.1 Lmx1b and FoxP2 in rats

**FIGURE 1:**
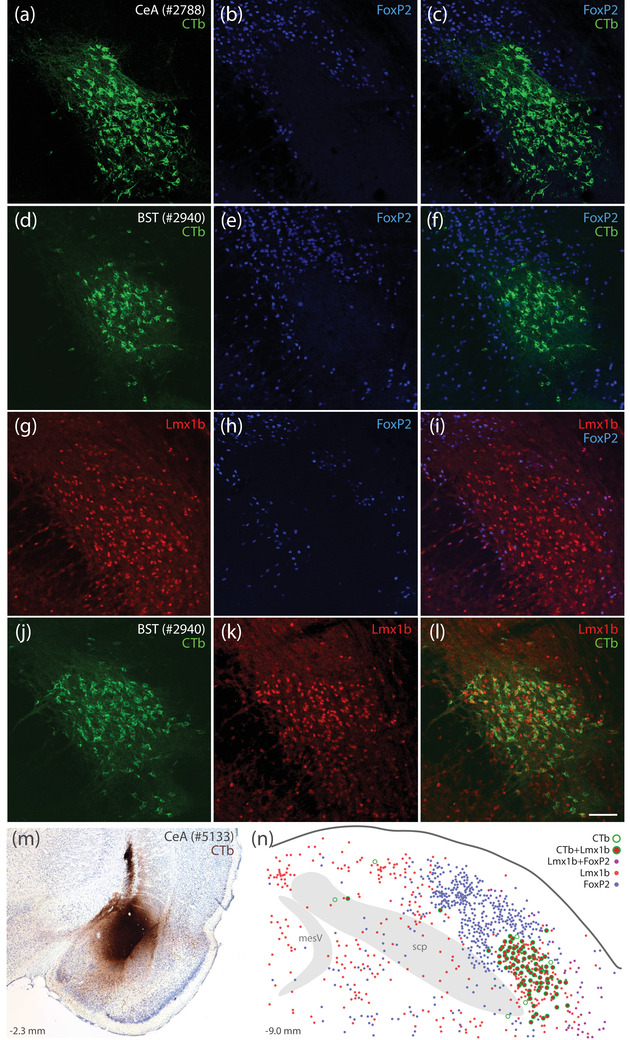
Lmx1b and FoxP2 in the rat parabrachial nucleus (PB). Neurons containing cholera toxin b (CTb, green) retrograde labeling after CTb injections into the central nucleus of the amygdala (CeA; a–c) or bed nucleus of the stria terminalis (BST; d–f) did not contain the transcription factor FoxP2 (blue). Neurons containing the transcription factor Lmx1b (red) filled a gap in the FoxP2 distribution (g–i) and were retrogradely labeled after CTb injection into the BST (j–l). Similarly, CTb injection into the CeA (m, rat case #5133) produced retrograde labeling predominantly in Lmx1b‐containing neurons (n). Approximate level caudal to bregma (in mm) is shown at bottom‐left in (m, n). Scale bar is 100 μm and applies to panels (a–l). Abbreviations: mesV, mesencephalic tract and nucleus of the trigeminal nerve; scp, superior cerebellar peduncle

Our investigation began in rats after identifying a small subpopulation of neurons activated by sodium deprivation (Geerling & Loewy, 2007). These sodium‐deprivation‐activated neurons intermingled with other PB neurons in a distribution that did not conform to existing, cytoarchitectural boundaries. Seeking a molecular marker that would allow us to better classify and then target these neurons, we examined regional expression of transcription factors and identified FoxP2 as a useful marker for these and several other PB subpopulations (Gasparini et al., 2021; Geerling et al., 2016; Geerling et al., 2011; Geerling et al., 2017; Shin et al., 2011; Verstegen et al., 2017). Virtually all PB neurons activated by sodium deprivation contained FoxP2 (Gasparini et al., 2021; Geerling et al., 2011), and many of these distributed within and alongside the superior cerebellar peduncle, in a location described as the inner portion of the external lateral PB subnucleus (PBeL; Herbert & Saper, 1990). Therefore, we predicted that sodium‐deprivation‐activated FoxP2 neurons, like other PBeL neurons, project axons to the bed nucleus of the stria terminalis and amygdala (Geerling & Loewy, 2006a, 2006b). To our surprise, retrograde tracer injections in each target region exclusively labeled PB neurons that did not contain FoxP2 (Figure 1a–f).

Seeking additional markers to fill this gap in the FoxP2 distribution led us to another transcription factor expressed in this region, Lmx1b (Asbreuk et al., 2002; Dai et al., 2008). Immunolabeling Lmx1b identified most neurons in PBeL, surrounded by a complementary distribution of neurons containing FoxP2 (Figure 1g–i). Lmx1b identified virtually all PB neurons that project axons to the bed nucleus of the stria terminalis (*n* = 2, Figure 1j–l) and central nucleus of the amygdala (*n* = 2, Figure 1m,n). Together, Lmx1b and FoxP2 labeling filled most of the PB, and their complementary distributions formed the basis of a study examining patterns of Fos activation after changes in blood pressure (Miller et al., 2012). These mutually exclusive distributions suggested the possibility of using transcription factors to classify all PB neurons. To explore this possibility using a richer set of genetic tools, we began studying the PB in mice.

### 3.2 Lmx1b and Foxp2 in mice

**FIGURE 2:**
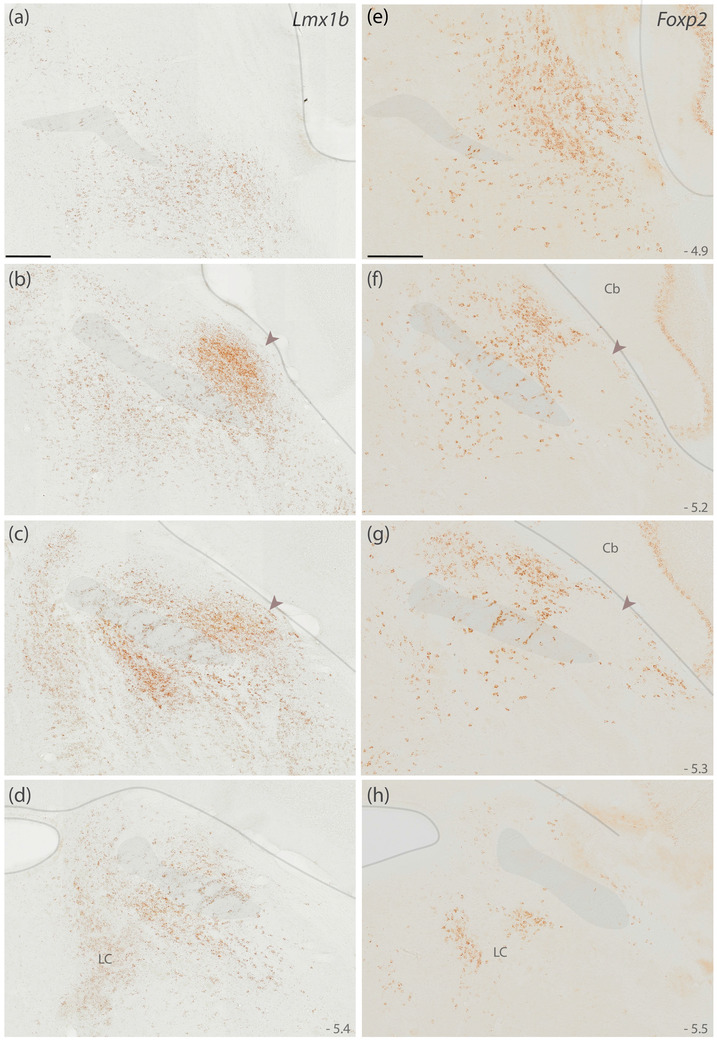
*Lmx1b* and *Foxp2* mRNA in the mouse PB. Diaminobenzidine (DAB) in situ hybridization revealed separate distributions of *Lmx1b* mRNA (a–d) and *Foxp2* mRNA (e–h) expression across four rostral‐to‐caudal sections through the PB region in mice. Approximate level caudal to bregma is shown at the bottom‐right of each panel (in mm). Translucent highlights identify the scp, brainstem surface, and fourth ventricular surface. Arrowheads in panels (b, c, f, g) highlight dense *Lmx1b* and absent *Foxp2* in a region of the mouse PB that is homologous to the “external lateral” subnucleus in rats (Fulwiler & Saper, 1984). Scale bars in (a) and (e) are 200 μm and apply to all panels below. Abbreviations: Cb, cerebellum; LC, locus coeruleus

First, to clarify the adult distribution of neurons expressing these two transcription factors in mice, we used brightfield in situ hybridization to separately label *Lmx1b* and *Foxp2* mRNA (Figure 2). Their distributions overlapped substantially, but *Lmx1b* skewed ventrally and medially, while *Foxp2* skewed dorsally and laterally. *Lmx1b* was most prominent in a region homologous to the rat PBeL, which was surrounded by dense *Foxp2* labeling. The medial PB contained a blend of both *Lmx1b* and *Foxp2*, each identifying cells scattered among and through fascicles of the superior cerebellar peduncle. *Lmx1b* labeling was more prominent caudally, but at far‐caudal levels of the PB, dense *Foxp2* labeling extended medially, through the locus coeruleus (LC), which contained labeling for *Lmx1b*. Lateral to the PB, we also found *Foxp2* labeling throughout the Purkinje layer of the cerebellum.

At these levels of the brainstem, *Lmx1b* labeling was densest in the raphe nuclei (not shown), and ventral to the PB, the supratrigeminal region and principal sensory trigeminal nucleus contained an extensive distribution of lighter *Lmx1b* labeling. This lighter *Lmx1b* expression in the supratrigeminal region extended ventrally, around the motor trigeminal nucleus, and caudally, into the hindbrain reticular formation. Within the foramen of Luschka, we also found *Lmx1b* labeling in a subset of choroid plexus epithelial cells (not shown).

**FIGURE 3:**
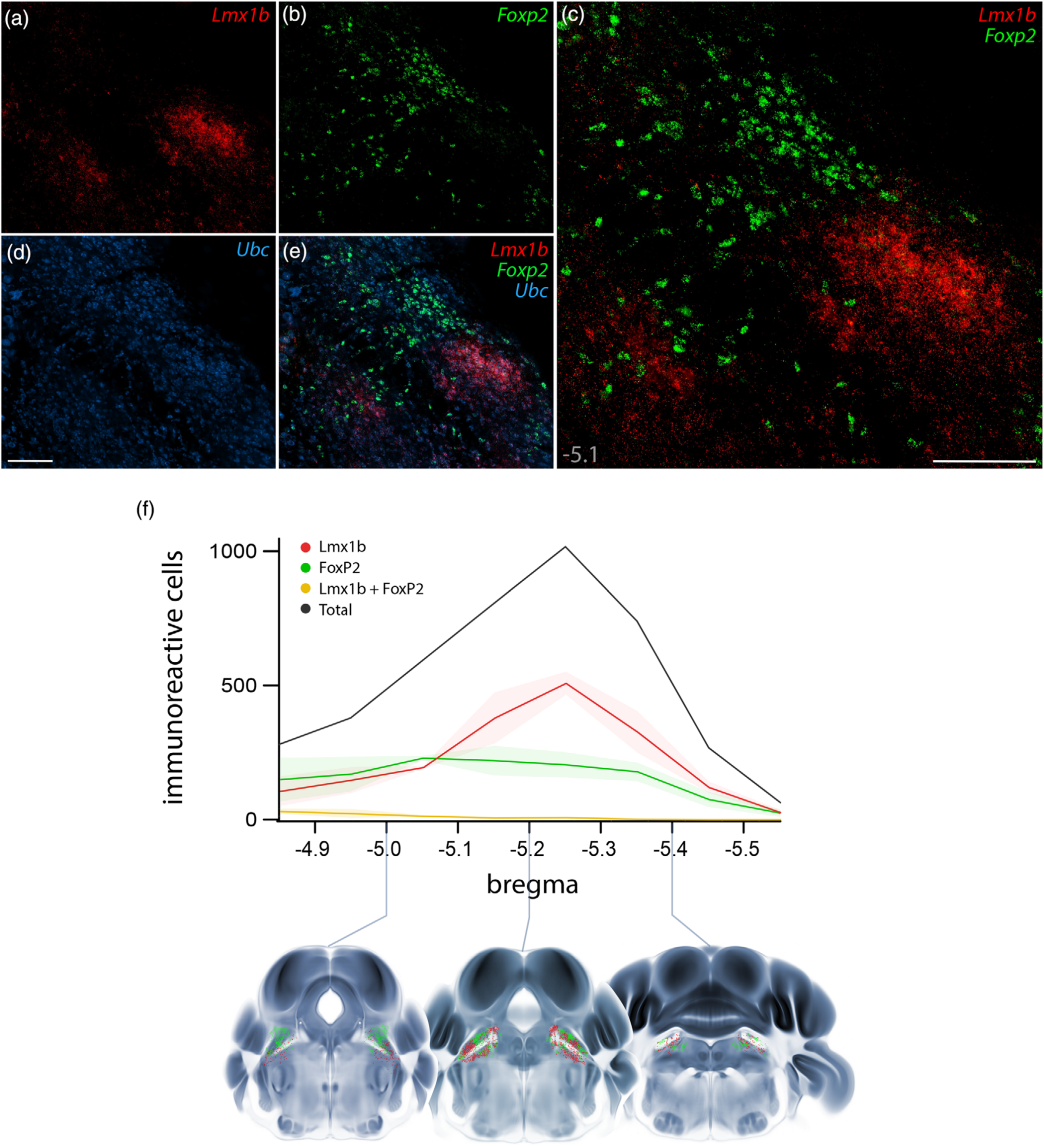
Fluorescence in situ hybridization (FISH) for *Lmx1b* and *Foxp2* mRNA and rostral‐to‐caudal counts of PB neurons containing Lmx1b and FoxP2 protein. Combined FISH labeling revealed the complementary expression patterns of *Lmx1b* mRNA (a, red) and *Foxp2* mRNA (b, green) at a mid‐level of the mouse PB (approximately bregma −5.1 mm). The ubiquitously expressed transcript *Ubc* (d, blue) was labeled for neuroanatomical background. Scale bars in (c) and (d) are 200 μm, and the scale bar in (d) applies to remaining panels (a, b, e). (f) After immunofluorescence labeling (see examples in Figure 4), average counts of neurons containing Lmx1b and FoxP2 are shown at each level (*n* = 3 mice), with variance represented by a standard deviation envelope. Approximate bregma levels are shown on the *x*‐axis, with three representative plots shown atop axial images from the Allen CCFv3 atlas (Wang et al., 2020)

Fluorescence in situ hybridization (FISH) labeling for *Foxp2* and *Lmx1b* mRNA confirmed their largely complementary distributions (Figure 3a–e). Per‐cell labeling for *Foxp2* mRNA was modest, but the small amount of *Lmx1b* mRNA per cell and the tight clustering of these cells in PBeL made it difficult to distinguish individual neurons. To more confidently identify and compare individual neurons, we switched to immunofluorescence labeling for Lmx1b and FoxP2.

**FIGURE 4:**
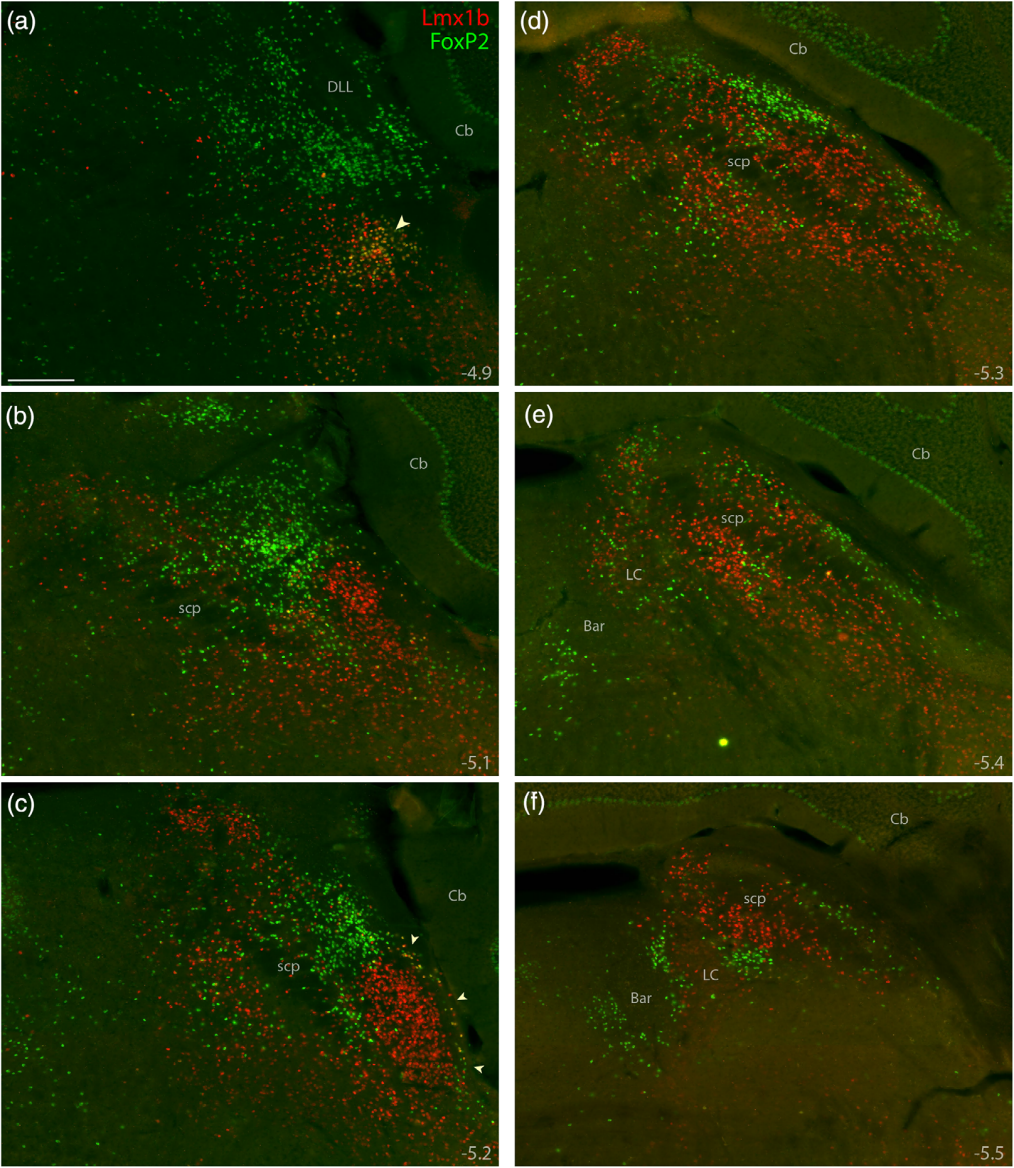
Immunofluorescence labeling for Lmx1b and FoxP2 protein. Combined immunolabeling for the nuclear transcription factors Lmx1b (red) and FoxP2 (green) identified largely separate distributions across successive, rostral‐to‐caudal sections through the PB region (a–f). Approximate level caudal to bregma is shown at the bottom‐right of each panel (in mm). Lmx1b immunofluorescence labeling was mutually exclusive with FoxP2, except in a rostral cluster of neurons ventral to the PB (arrowhead in a) and in sparse, double‐labeled neurons extending back to mid‐rostral levels of the PB along the lateral edge of the brainstem (arrowheads in c). Scale bar in (a) is 200 μm and applies to panels (b–f). Abbreviations: Bar, Barrington's nucleus; DLL, dorsal nucleus of the lateral lemniscus

Robust immunofluorescence labeling (Figure 4) matched the patterns of mRNA labeling described above, except that Lmx1b immunoreactivity was less intense than *Lmx1b* mRNA labeling in LC neurons. Quantitative analysis across all rostrocaudal levels of the PB revealed that most immunolabeled neurons (98%) contained either Lmx1b or FoxP2, not both (*n* = 3 mice, Figure 3f).

However, at rostral levels and along the ventrolateral margin of the PB, a small subset contained both Lmx1b and FoxP2. This double‐labeled subset––roughly 2% of the combined total––clustered primarily within the KF (Figure 4a), as in rats (Miller et al., 2012). These double‐labeled neurons intermingled with many others that contained Lmx1b alone. This double‐labeled population also extended caudally along the edge of the brainstem, outside PBeL (Figure 4c), in a distribution resembling the “lateral crescent” in rats (Chamberlin & Saper, 1992; Miller et al., 2012).

### 3.3 Glutamatergic and GABAergic subsets

**FIGURE 5:**
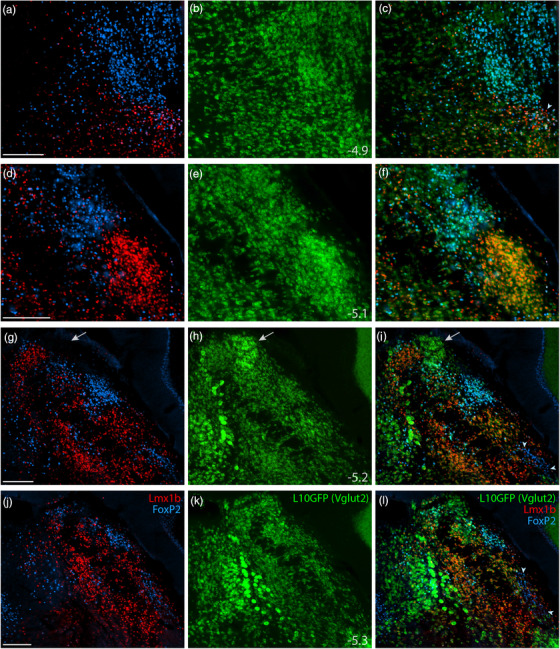
Lmx1b and FoxP2 in a glutamatergic Cre‐reporter mouse. Immunofluorescence labeling identified Lmx1b (red) and FoxP2 (blue) in the PB of mice expressing an L10GFP Cre‐reporter (green) for the glutamatergic marker gene *Slc17a6* (vesicular glutamate transporter 2, Vglut2). Arrowhead in (c) indicates a cluster of triple‐labeled neurons (Lmx1b+FoxP2+L10GFP) located rostrally, in the KF region. White arrows in (g–i) highlight a dorsal cluster of L10GFP‐expressing neurons lacking both Lmx1b and FoxP2. Arrowheads in (i) and (l) indicate a ventrolateral cluster of neurons labeled for FoxP2 in the “caudal KF.” Approximate bregma levels are shown at bottom‐right in the center column (in mm). All scale bars are 200 μm and apply to other panels in their respective rows

**FIGURE 6:**
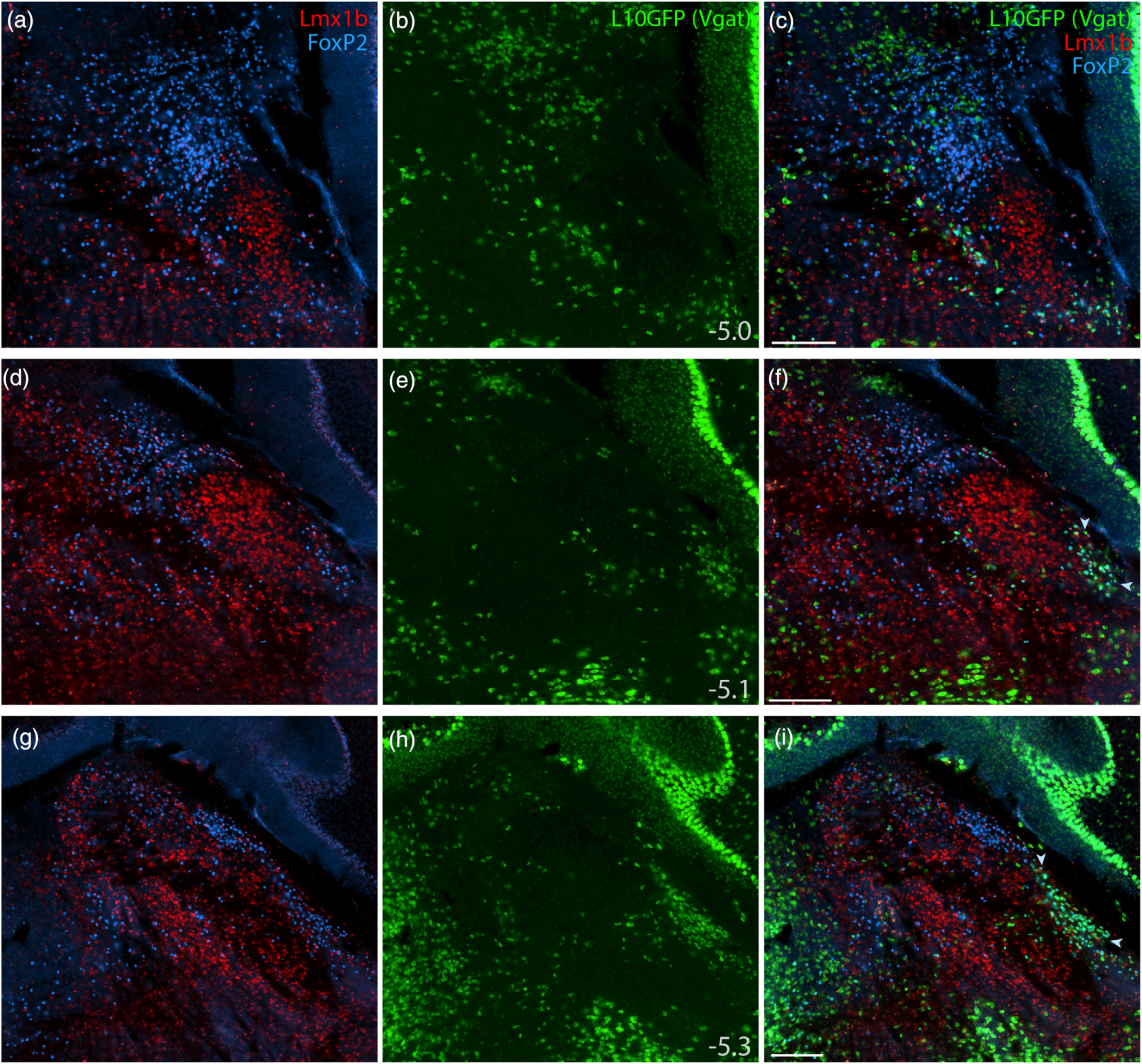
Lmx1b and FoxP2 in a GABAergic Cre‐reporter mouse. Immunofluorescence labeling identified Lmx1b (red) and FoxP2 (blue) at three rostral‐to‐caudal levels of the PB in mice expressing an L10GFP Cre‐reporter (green) mice for the GABAergic marker gene *Slc32a1* (vesicular GABA transporter, Vgat). Arrowheads in panels (f) and (i) indicate a caudal, ventrolateral cluster of neurons that contain FoxP2 and express L10GFP in the “caudal KF.” Approximate bregma levels are shown at bottom‐right in the center column (in mm). All scale bars are 200 μm and apply to other panels in their respective rows

GABAergic neurons flank the PB on several sides (Geerling et al., 2017; Li et al., 2005; Verstegen et al., 2017), and a recent study reported that 12% of neurons in this region are GABAergic (Raver et al., 2020), so it was important to clarify whether neurons identified by Lmx1b and FoxP2 are excitatory (glutamatergic; *Slc17a6*/Vglut2‐expressing) or inhibitory (GABAergic; *Slc32a1*/Vgat‐expressing). To do this, we used separate Cre‐reporter strains for Vglut2 and Vgat (Figures 5 and 6). In each strain, we used a GFP Cre‐reporter that is tethered to the L10 ribosomal subunit (L10GFP), which concentrates in the cell body, maximizing our ability to distinguish neurons and colocalize other markers. The complementary distributions of L10GFP‐expressing neurons between Vglut2 and Vgat strains matched our previous descriptions (Geerling et al., 2016; Geerling et al., 2017; Huang et al., 2020). In addition to sparse GABAergic neurons scattered through the PB, we observed flanking GABAergic populations medially, in the pontine central gray, and ventrolaterally, in a population we have referred to as the “caudal KF” (Geerling et al., 2017).

All Lmx1b‐labeled neurons in the PB and KF expressed L10GFP in glutamatergic Cre‐reporter mice (Figure 5), and no Lmx1b‐labeled neurons expressed L10GFP in the GABAergic strain (Figure 6). FoxP2‐labeled neurons in PB and KF also colocalized with L10GFP in glutamatergic Cre‐reporter mice, but a caudal cluster of FoxP2‐labeled neurons ventrolateral to the PB (“caudal KF”) expressed L10GFP exclusively in GABAergic reporter mice (arrowheads in Figures 5i,l and 6f,i). Some FoxP2‐labeled GABAergic neurons distributed rostrally, intermingling with the caudal aspect of the glutamatergic KF population. Ventrolateral to the PB, all neurons that contained FoxP2 and lacked Lmx1b expressed L10GFP exclusively in GABAergic reporter mice. Conversely, all neurons in the rostral population containing both Lmx1b and FoxP2 expressed L10GFP exclusively in glutamatergic reporter mice. Outside the PB, FoxP2 neurons in the pontine central gray matter (ventromedial to Barrington's nucleus) and cerebellar Purkinje neurons expressed L10GFP exclusively in GABAergic reporter mice (Figure 6g–i).

At mid‐levels of the PB, Vglut2 Cre‐reporter labeling identified an additional, dorsal cluster of neurons lacking both Lmx1b and FoxP2 (arrow in Figure 5g–i). This prominent cluster resembled the rat “internal lateral” PB subnucleus (Bester et al., 1999; Bourgeais et al., 2001; Feil & Herbert, 1995; Fulwiler & Saper, 1984; Krout & Loewy, 2000).

### 3.4 Phox2b ventral to the PB

The ventral gradient of lighter *Lmx1b* expression (Figure 2) left an indefinite distinction between the PB and several other brainstem nuclei, so we sought a molecular marker that could distinguish PB neurons from underlying populations. Without imposing any cytoarchitectural or other external constraints, we found that neurons ventral to the PB can be distinguished by labeling another transcription factor that had been identified in this brainstem region in rats, Phox2b (Kang et al., 2007).

**FIGURE 7:**
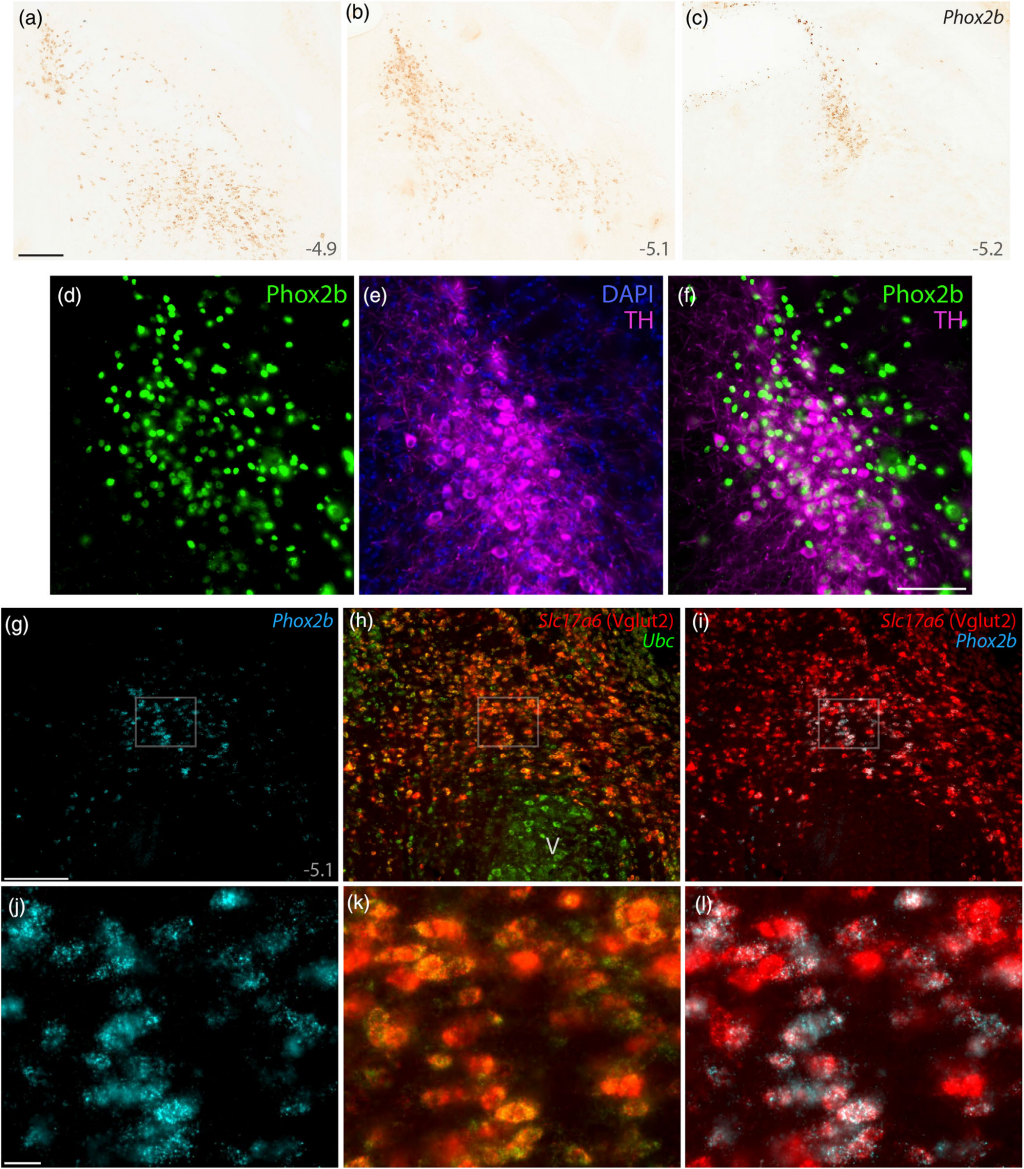
*Phox2b* identifies glutamatergic and catecholaminergic populations ventral and medial to the PB. DAB in situ hybridization revealed *Phox2b* mRNA, shown at three rostral‐to‐caudal levels (a–c). Approximate level caudal to bregma is shown at the bottom‐right of each panel (in mm). (d–f) Immunofluorescence labeling for Phox2b (green) and tyrosine hydroxylase (TH, magenta) identify LC neurons. (g–i) FISH identified colocalization between *Phox2b* mRNA (ice‐blue) and the glutamatergic marker *Slc17a6* mRNA (h, red) ventral to a mid‐level of the PB (bregma −5.1 mm). *Ubc* mRNA (green) is shown for neuroanatomical background. (j–l) Blow‐ups of the highlighted region in (g–i) show the ubiquitous colocalization of *Slc17a6* mRNA in *Phox2b*‐expressing neurons in the supratrigeminal region. All scale bars are 200 μm and apply to related panels. Abbreviation: V, motor trigeminal nucleus

**FIGURE 8:**
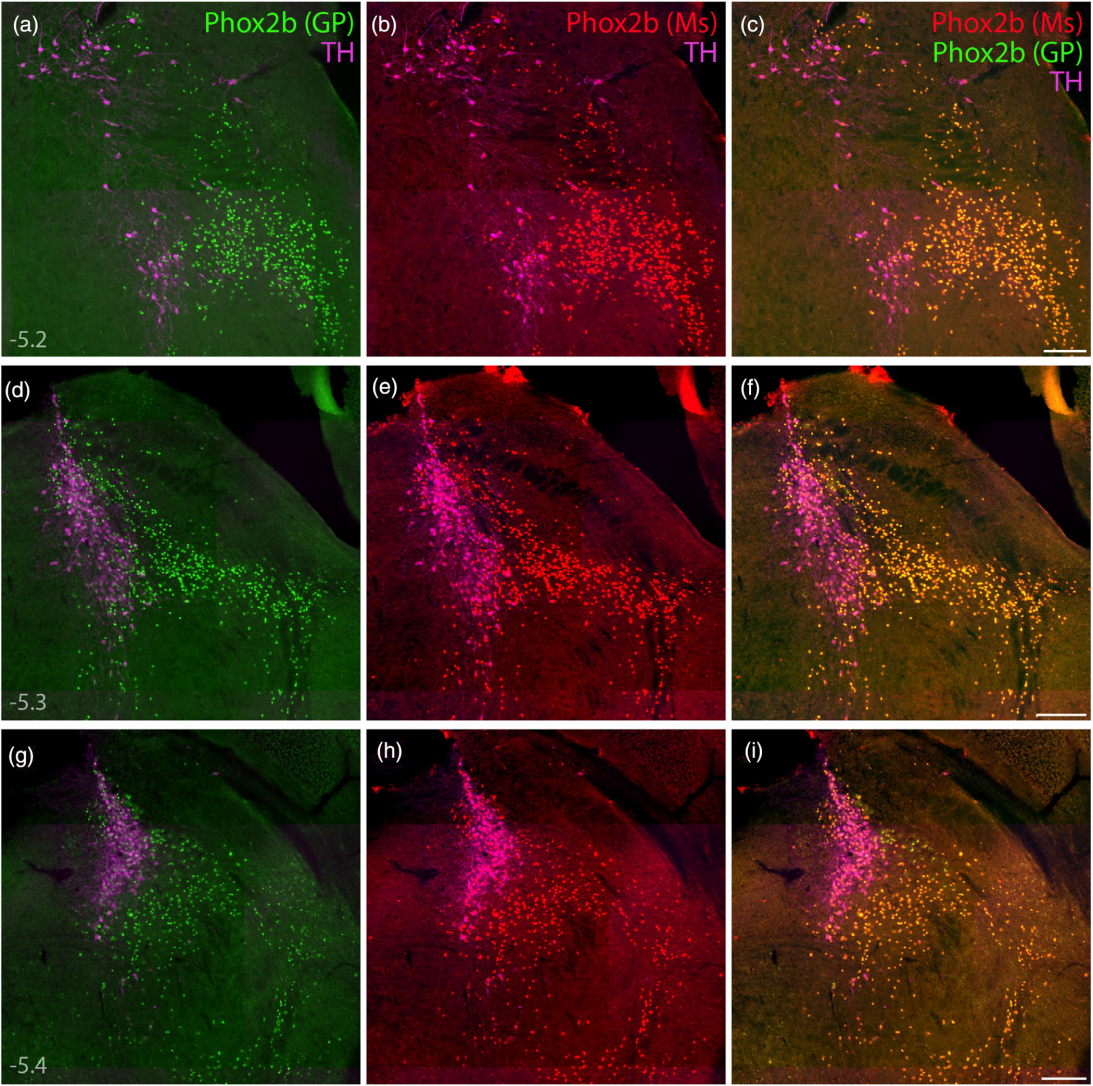
Immunofluorescence labeling for Phox2b. (a–i) Direct comparison of immunofluorescence labeling between guinea pig polyclonal [green, “Phox2b (GP)”; (Nagashimada et al., 2012)] and mouse monoclonal [red, “Phox2b (Ms)”; sc‐376997] anti‐Phox2b antisera, combined with TH immunofluorescence (magenta) to identify LC neurons. In addition to confirming antibody specificity, labeling Phox2b across three rostral‐to‐caudal levels of the PB region highlighted the extensive population of non‐LC (glutamatergic) Phox2b neurons, which form an observer‐independent ventromedial border for the PB. Approximate bregma levels are shown at bottom‐left in (a, d, g). Scale bars in (c, f, i) are 200 μm, and each scale bar applies to other panels in the same row

*Phox2b* mRNA labeling (Figure 7a–c) and Phox2b immunofluorescence (Figure 8) identified a large distribution of neurons immediately ventral to the PB. At mid‐levels, the dorsal aspect of this population formed a sharp, ventromedial border for the PB. The PB itself contained very few *Phox2b*‐expressing cells. Just medial to the PB, mRNA labeling and Phox2b immunoreactivity were prominent in and around the LC. Everywhere in this region, Phox2b was mutually exclusive with FoxP2. *Phox2b*‐expressing cells occupied much of the supratrigeminal region that divides the PB from the trigeminal motor and principal sensory nuclei (both of which lacked *Phox2b* mRNA labeling and lacked Phox2b immunofluorescence). Like the *Lmx1b* distribution, contiguous *Phox2b* labeling extended ventrally and caudally from the supratrigeminal region into the hindbrain reticular formation.

The supratrigeminal region contains both excitatory and inhibitory neurons. Vgat Cre‐reporter labeling, mRNA labeling for Vgat (*Slc32a1*), and mRNA labeling for the glycinergic neuronal marker *Slc6a5* identified large neurons caudally and many smaller neurons rostrally in this region (not shown). Also, Vglut2 Cre‐reporter labeling and mRNA labeling for *Slc17a6* identified many small neurons in the supratrigeminal region (not shown). We used FISH to determine which population expresses *Phox2b* and found extensive *Slc17a6* mRNA labeling in small *Phox2b*‐expressing neurons (Figure 7g–l), but not in larger *Phox2b*‐expressing neurons in the LC. Thus, in addition to nonglutamatergic LC neurons, *Phox2b* identifies a large population of glutamatergic neurons in the supratrigeminal nucleus and reticular formation, immediately ventral to the PB.

Medial to the PB, the dorsomedial distribution of this glutamatergic *Phox2b* population intermingled through the mesencephalic trigeminal nucleus and the dorsal LC. The LC contained both *Phox2b* mRNA labeling and Phox2b immunolabeling. Every LC neuron had cytoplasmic immunofluorescence labeling for tyrosine hydroxylase surrounding a Phox2b‐immunolabeled nucleus, but these intermingled with a population of smaller, noncatecholaminergic neurons with more intense nuclear immunofluorescence for Phox2b (Figure 7d–f). The PB contained fewer *Phox2b*‐expressing neurons, which were identical in appearance (and *Slc17a6* labeling; not shown) to the smaller glutamatergic neurons ventral and medial to it and appeared to be the fringe of the supratrigeminal population, rather than a distinct subpopulation of PB neurons.

**FIGURE 9:**
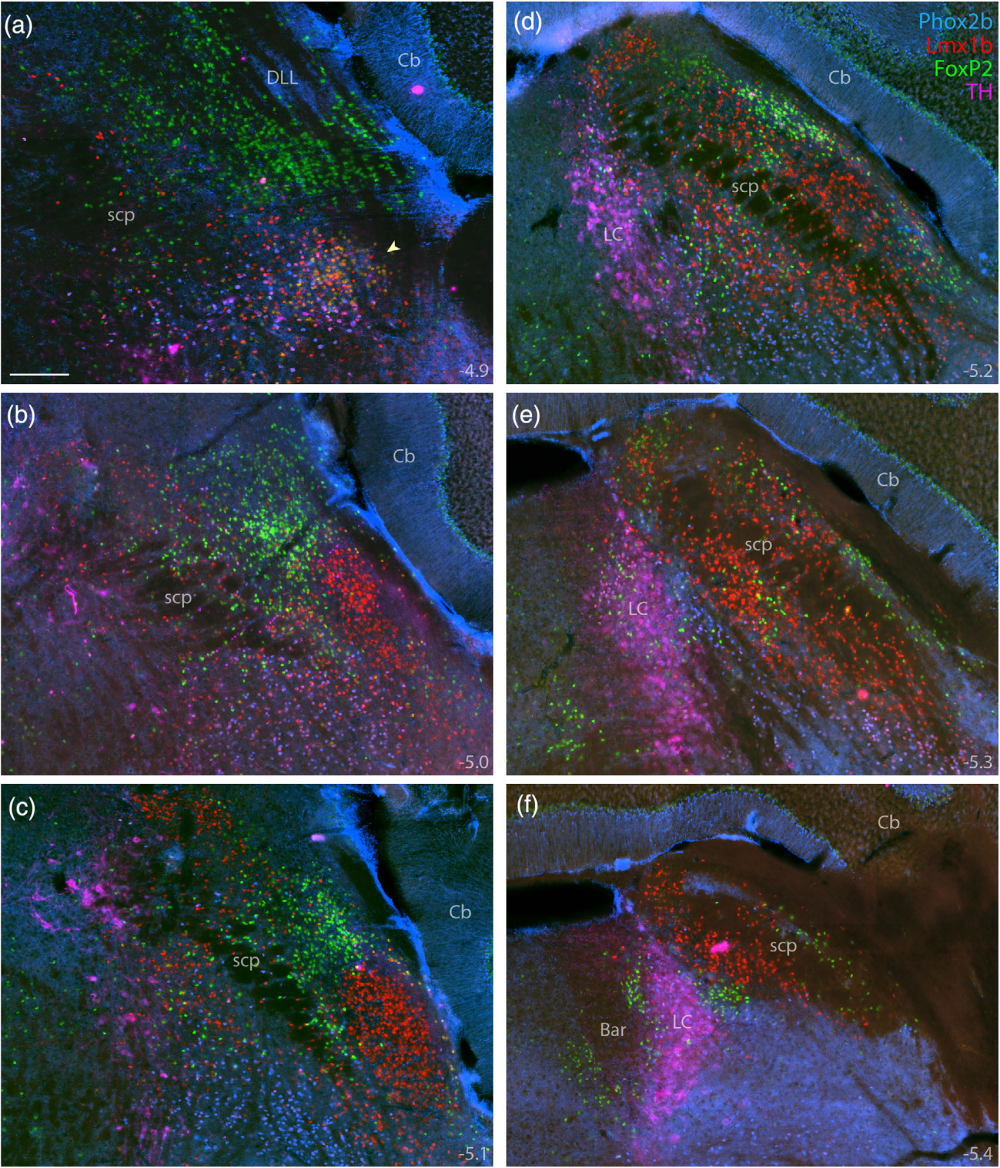
Combined immunofluorescence labeling for Phox2b, Lmx1b, and FoxP2. Immunolabeling for Phox2b (blue, mouse monoclonal antibody) combined with Lmx1b (red) and FoxP2 (green), across six rostral‐to‐caudal sections through the PB region (a–f), distinguished adult PB neurons from underlying non‐PB populations. Additional labeling for TH (magenta) distinguished Phox2b neurons in the LC from those in the supratrigeminal population. Approximate bregma levels are shown at the bottom‐right of each panel. Arrowhead in (a) highlights the KF. Scale bar in (a) is 200 μm and applies to all panels

**FIGURE 10:**
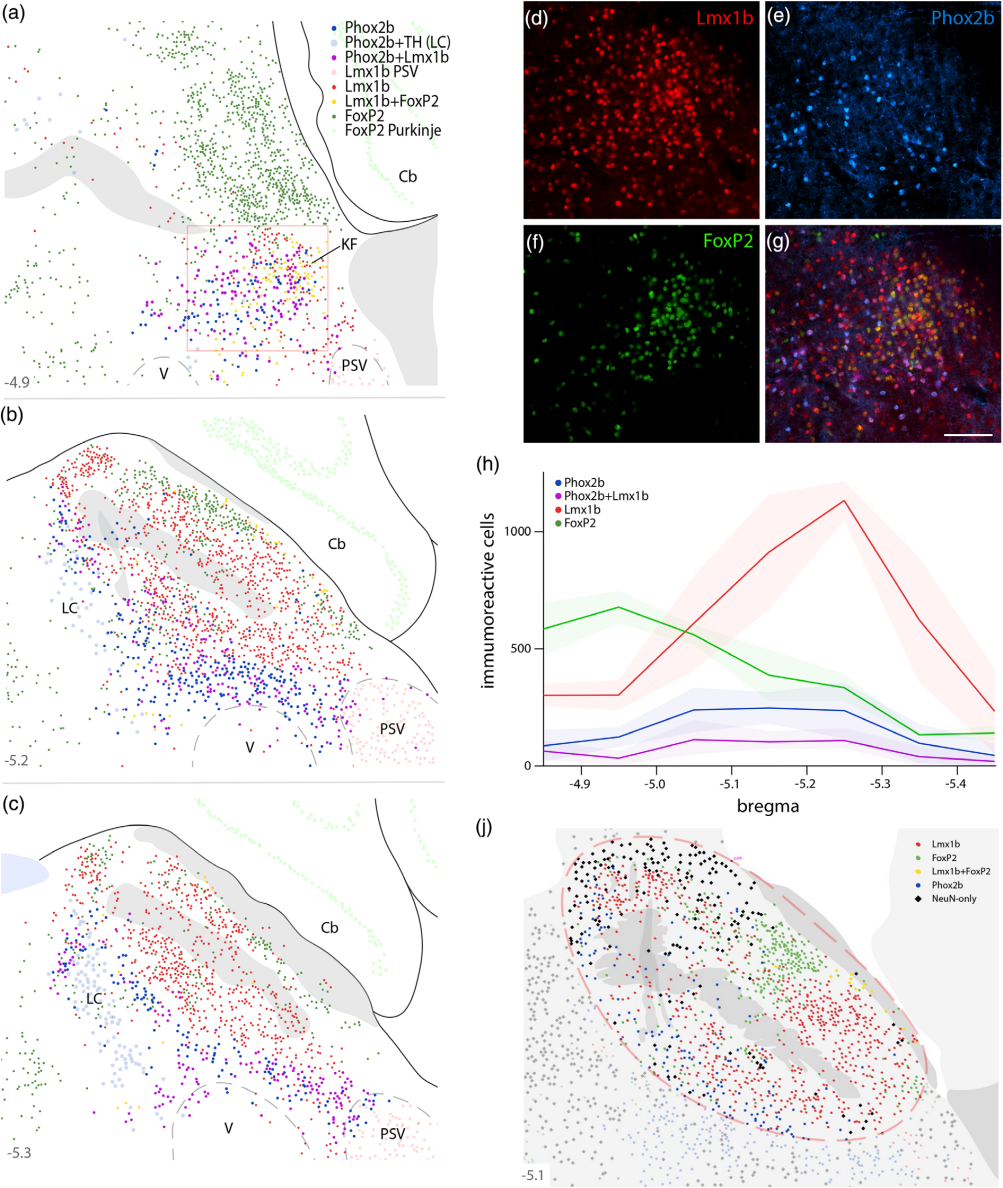
Labeling Phox2b, along with Lmx1b and FoxP2, identifies diverse populations of adult neurons in and around the PB. Neurons containing Phox2b alone (blue), Phox2b+Lmx1b (magenta), Lmx1b alone (red), Lmx1b+FoxP2 (yellow), or FoxP2 alone (green) were plotted at three rostral‐to‐caudal levels of the mouse PB (a–c). Approximate level caudal to bregma is shown at the bottom‐left of each panel (in mm). Phox2b‐containing neurons in the LC (TH+Phox2b) were plotted in light blue. FoxP2‐containing Purkinje neurons in the cerebellum were plotted in light green. Large, Lmx1b‐containing neurons in the principal sensory trigeminal nucleus (PSV) were plotted in light red. Ventral to the rostral PB (box in a), immunofluorescence labeling for Lmx1b (d, red), Phox2b (e, blue), FoxP2 (f, green), and all three combined (g) revealed focally diverse populations in the KF region. Scale bar in (g) is 50 μm. (h) Rostral‐to‐caudal counts of PB neurons containing Phox2b alone, Lmx1b alone, FoxP2 alone, or Phox2b+Lmx1b were averaged at each level (*n* = 3 mice), with variance represented by a standard deviation envelope; approximate levels caudal to bregma are labeled on the *x*‐axis. (i) Plotting the distribution of neurons containing only NeuN (black diamonds), without Phox2b, Lmx1b, or FoxP2, highlighted a remaining set of unidentified PB neurons. Approximate bregma level is shown at the bottom‐left of each panel (in mm)

The *Phox2b* distribution substantially overlapped the gradient of lighter *Lmx1b* labeling ventral to the PB. At first, we were unable to determine whether any neurons contained both Phox2b and Lmx1b because both antisera were raised in the same species (guinea pig), but serial‐labeling protocols (for Lmx1b then Phox2b; and, separately, for Phox2b then Lmx1b) revealed a large population of potentially coexpressing neurons. To better assess colocalization, we identified a mouse monoclonal antibody that labels Phox2b. Double‐labeling tissue with this antibody (sc‐376997) along with the guinea pig anti‐Phox2b (Nagashimada et al., 2012) highlighted an identical set of neurons (Figure 8). Next, using this mouse monoclonal in combination with antisera for Lmx1b (guinea pig), FoxP2 (sheep), and TH (rabbit) confirmed that many supratrigeminal neurons contain both Phox2b and Lmx1b (Figures 9 and 10). Lmx1b labeling here (ventral to the PB) was lighter and always colocalized with Phox2b, while Lmx1b labeling in the PB was more intense and rarely colocalized with Phox2b. Across all rostrocaudal levels, we found Lmx1b immunofluorescence in approximately half the neurons that contained Phox2b (range 44–57%, *n* = 3 mice), but given the light and variable intensity of Lmx1b labeling in the supratrigeminal region, this may be an underestimate.

### 3.5 Diverse Kölliker–Fuse subpopulations

Rostrally and laterally, the Phox2b population merged seamlessly through the KF (Figures 9a and 10a). This was unexpected because a previous study in rats found Phox2b in very few KF neurons (Kang et al., 2007). Including this substantial Phox2b‐immunoreactive population, the KF contained the greatest transcription factor diversity in this region of the brainstem.

As noted above, most neurons in the KF region contained Lmx1b. Within that population, mutually exclusive subsets contained either FoxP2 or Phox2b, never both (Figure 10a, d–g). Many, intermingled neurons contained only Lmx1b (without Phox2b or FoxP2), and this subset intermingled with a sizeable minority of KF neurons that contained only Phox2b (without Lmx1b). These findings highlight the KF and contiguous supratrigeminal region as an intersection of multiple glutamatergic populations. These include: (1) an *Lmx1b*‐derived population resembling “dA3” hindbrain interneurons, which also express *Phox2b* (Gray, 2013; Hernandez‐Miranda et al., 2017), (2) a distinctly KF subpopulation containing both *Lmx1b* and *Foxp2* (without *Phox2b*), (3) an intermingled population containing just *Lmx1b* (without *Phox2b* or *Foxp2*), and (4) an intermingled population containing just *Phox2b* (without *Foxp2* or *Lmx1b*). Also, along the caudal aspect of this region are (5) GABAergic, *Foxp2*‐expressing neurons (“caudal KF”), some of which extend rostrally beneath the PB and intermingle with the caudal aspect of the atlas‐defined KF region.

### 3.6 Unidentified neurons

To determine whether the PB region includes additional neurons that were not identified by our adult transcription factor markers (Lmx1b, FoxP2, and Phox2b), we labeled all three in combination with a more broadly expressed neuronal marker, NeuN (encoded by the transcription factor *Rbfox3*; Kim et al., 2009). Besides LC neurons, which lack NeuN (Verstegen et al., 2017), all FoxP2‐, Lmx1b‐, and Phox2b‐immunoreactive neurons in the PB contained NeuN immunoreactivity. Conversely, most NeuN‐immunoreactive neurons contained at least one of these transcription factors. However, a substantial minority of NeuN‐immunoreactive neurons did not contain FoxP2, Lmx1b, or Phox2b. These “NeuN‐only” neurons were sparse ventrally and concentrated rostrally and dorsally in the lateral PB (Figure 10i), including the subregion with glutamatergic neurons lacking both FoxP2 and Lmx1b (Figure 5i). To identify these remaining neurons, we analyzed two other transcription factors that influence the development of neurons in this region––*Lmx1a* and *Atoh1*.

### 3.7 Lmx1a

First, we examined adult expression of *Lmx1a*, the paralog of *Lmx1b*. During embryogenesis, *Lmx1a* and *Lmx1b* play overlapping roles in the roof plate of the neural tube (Chizhikov et al., 2021; Liu et al., 2010; Mishima et al., 2009) and have partly overlapping expression patterns (Chen et al., 1998; Chizhikov et al., 2006; Chizhikov et al., 2010; Costa et al., 2001; Ding et al., 2004; Dreyer et al., 1998; Guo et al., 2007; Guo et al., 2008; Kuwamura et al., 2005; Millonig et al., 2000; Zhao et al., 2006). Also, these transcription factors are expressed by partly overlapping populations of neurons in the mature brain (Asbreuk et al., 2002; Dai et al., 2008; Zou et al., 2009).

**FIGURE 11:**
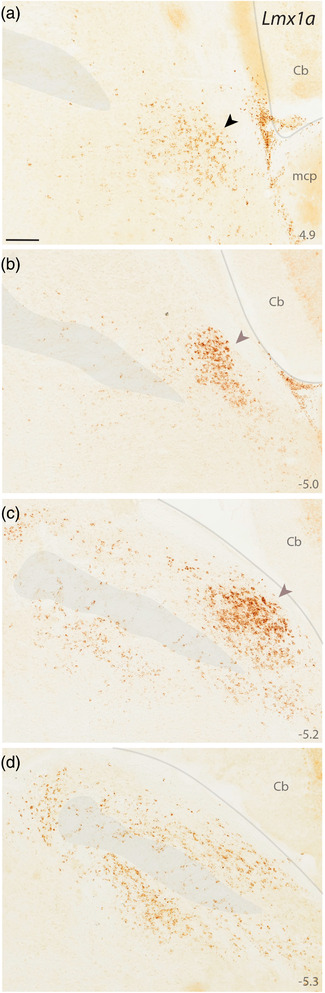
*Lmx1a* mRNA labeling. DAB in situ hybridization revealed the distribution of *Lmx1a* expression at four, successive rostral‐to‐caudal sections through the PB region. Approximate level caudal to bregma is shown at bottom right (in mm). Black arrowhead (a) highlights the KF region. Translucent highlights identify the scp and boundaries between the brainstem surface and cerebellum. Mauve arrowheads (b, c) highlight dense *Lmx1a* labeling in a region homologous to the “external lateral” subnucleus in rats (Fulwiler & Saper, 1984). Scales bar in (a) is 200 μm and applies to all panels. Abbreviation: mcp, middle cerebellar peduncle

In the adult PB, *Lmx1a* mRNA labeling resembled *Lmx1b* (Figure 11). Like *Lmx1b*, *Lmx1a* skewed ventrally (through PBeL and KF) and caudally (through the superior cerebellar peduncle and medial PB). Unlike *Lmx1b*, however, we did not find *Lmx1a* labeling in the LC. Nor was there any *Lmx1a* labeling in the supratrigeminal nucleus or principal sensory trigeminal nucleus. We found *Lmx1a* expression in several regions that lacked *Lmx1b*, including the cerebellar flocculus, superior vestibular nucleus, and dorsal cochlear nucleus (not shown), as well as a population of small, dense cells along the brainstem surface and atop the middle cerebellar peduncle (Figure 11a,b). Also, the choroid plexus had more extensive *Lmx1a* labeling than *Lmx1b* (not shown).

**FIGURE 12:**
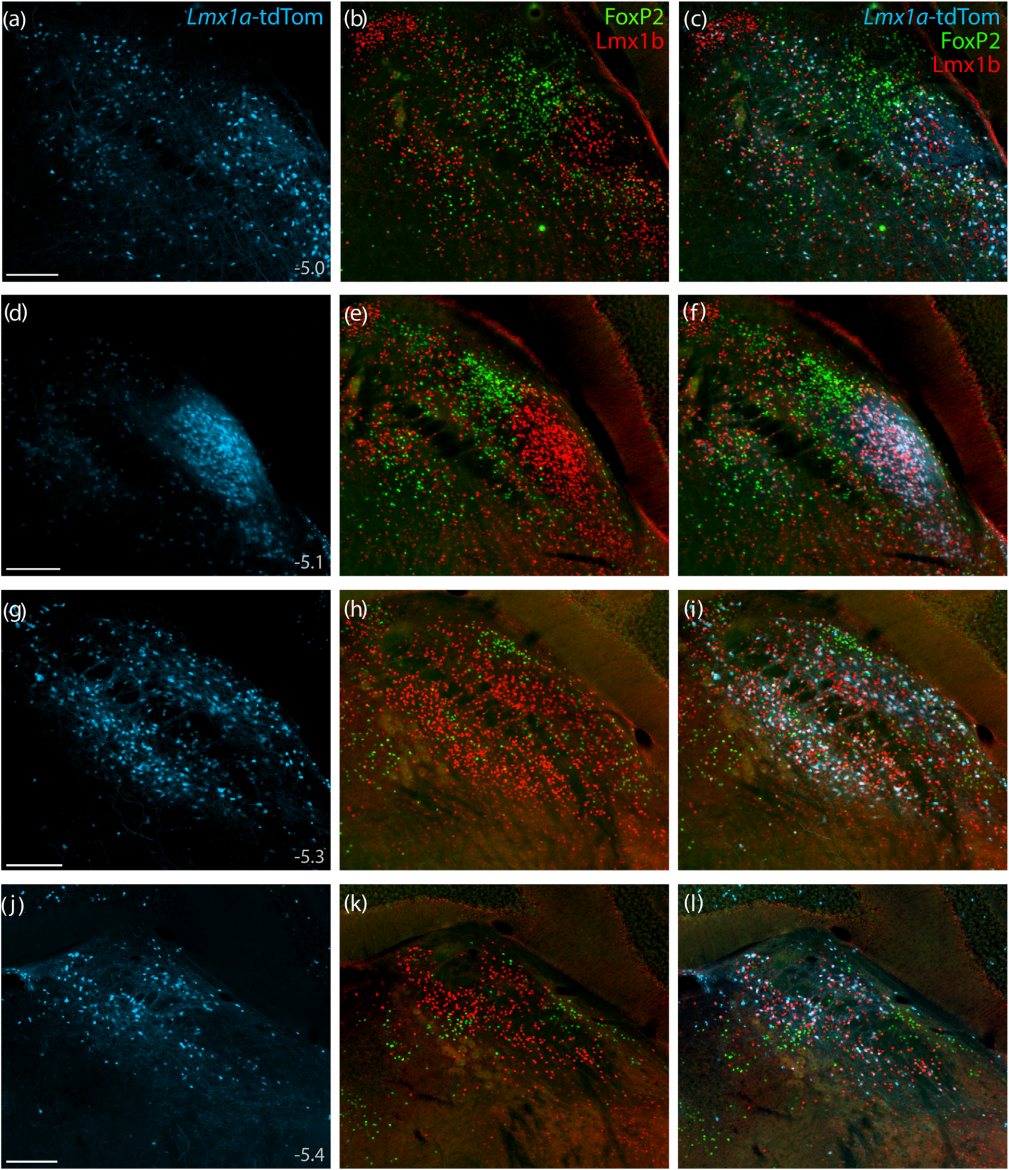
*Lmx1a* Cre fate‐mapping. Cre fate‐mapping for *Lmx1a* (tdTomato, pseudocolored ice blue), followed by immunofluorescence labeling for Lmx1b (red) and FoxP2 (green), shown at four rostral‐to‐caudal levels through the PB region. Approximate level caudal to bregma is shown at bottom‐left in the first panel of each row (in mm). Scale bars (a, d, g, j) are 200 μm and apply to all other panels in the same row

**FIGURE 13:**
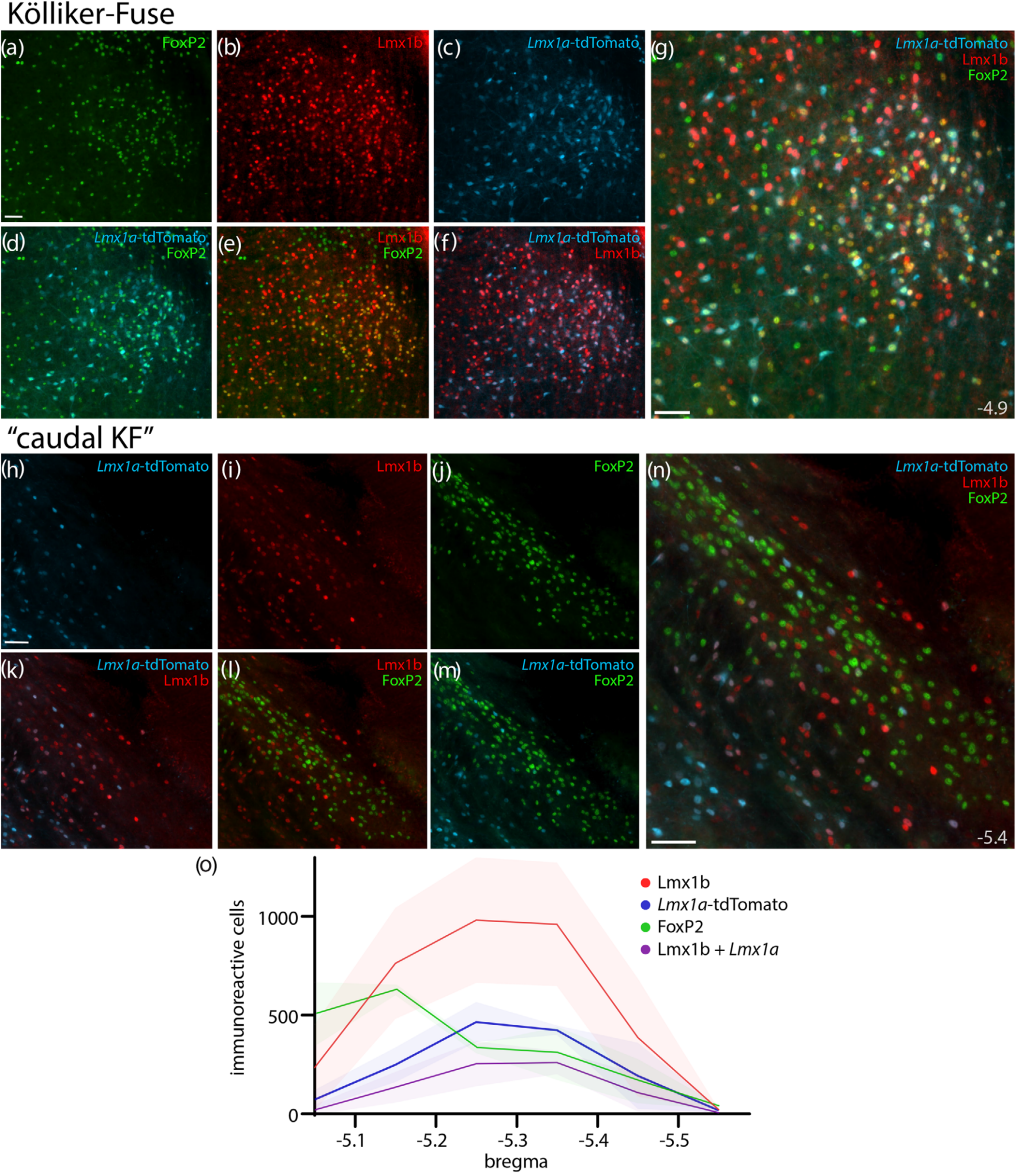
*Lmx1a*‐derived KF neurons and rostral‐to‐caudal counts across the PB region. Panels (a–g) show Cre fate‐mapping for *Lmx1a* (tdTomato, pseudocolored ice blue) and immunofluorescence labeling for Lmx1b (red) and FoxP2 (green) in the KF region (ventral to the rostral PB). Panels (h–n) show the lack of *Lmx1a* Cre‐reporter expression in “caudal KF” neurons that contain FoxP2 (ventrolateral to the caudal PB). Approximate level caudal to bregma (in mm) is shown at bottom‐right in (g, n). All scale bars are 50 μm. Scale bar in (a) applies to (b–f) and scale bar in (h) applies to (i–m). Bottom graph (o) shows rostral‐to‐caudal counts of PB neurons expressing the tdTomato Cre‐reporter for *Lmx1a*, FoxP2, or Lmx1b, as well as neurons containing both Lmx1b and *Lmx1a* Cre‐reporter. Counts were averaged at each level (*n* = 3 mice), with variance represented by a standard deviation envelope. Approximate bregma levels are shown on the *x*‐axis

As with *Lmx1b*, the low per‐cell content of *Lmx1a* mRNA made it difficult to distinguish individual neurons. Lacking an antibody that labels this transcription factor, we used Cre fate‐mapping to identify cells that have expressed *Lmx1a* or developed from *Lmx1a*‐expressing precursors. In *Lmx1a‐Cre*;*R26‐LSL‐tdTomato* mice (*n* = 5, Figure 12), the distribution of tdTomato expression resembled the distribution of *Lmx1a* mRNA. Most tdTomato‐expressing (putatively *Lmx1a*‐derived) PB neurons also contained Lmx1b (69 ± 8%, *n* = 3; graph in Figure 13o), but an intermingled minority lacked Lmx1b, and a larger population of Lmx1b‐containing neurons lacked tdTomato, consistent with the subtotal colocalization of *Lmx1a* mRNA and Lmx1b in P7 mouse pups (Figure 3J of Zou et al., 2009). These populations had similar, intermingled distributions, except for a few tdTomato‐expressing neurons that lacked Lmx1b caudal and medial to the PB (between the LC and fourth ventricle; not shown). Besides these, the population identified by *Lmx1a* Cre fate‐mapping included few neurons that were not identified already by Lmx1b and very few in the rostral‐dorsal distribution of “NeuN‐only” neurons we sought to identify.

Rostrally, many neurons expressed tdTomato in the KF region (Figure 13). All these putatively *Lmx1a*‐derived neurons contained Lmx1b, and many also contained FoxP2 (in addition to Lmx1b), but no other FoxP2‐labeled neurons in the PB or “caudal KF” expressed tdTomato. Outside the PB, LC neurons containing tyrosine hydroxylase did not express tdTomato. We also did not find tdTomato expression (or labeling for Lmx1b, FoxP2, or Phox2b) in the larger neurons containing choline acetyltransferase (ChAT) in the laterodorsal and pedunculopontine tegmental nuclei (not shown), although Lmx1b and tdTomato did colocalize with a small number of faintly ChAT‐immunoreactive neurons in the caudal PB and PBeL (not shown).

### 3.8 Atoh1 derivation identifies remaining PB neurons

After *Lmx1a* Cre fate‐mapping failed to identify many neurons in the “NeuN‐only” distribution, we next focused on neurons that derive from *Atoh1*‐expressing precursors. Across several days of embryonic development, *Atoh1*‐expressing precursors in the rhombic lip neuroepithelium produce (1) glutamatergic neurons that populate the PB and many other brainstem nuclei, followed by (2) glutamatergic neurons in the deep cerebellar nuclei, and then (3) the entire granule cell layer of the cerebellum (Ben‐Arie et al., 1997; Bermingham et al., 2001; Gray, 2008, 2013; Machold & Fishell, 2005; Rose et al., 2009; Wang et al., 2005).

**FIGURE 14:**
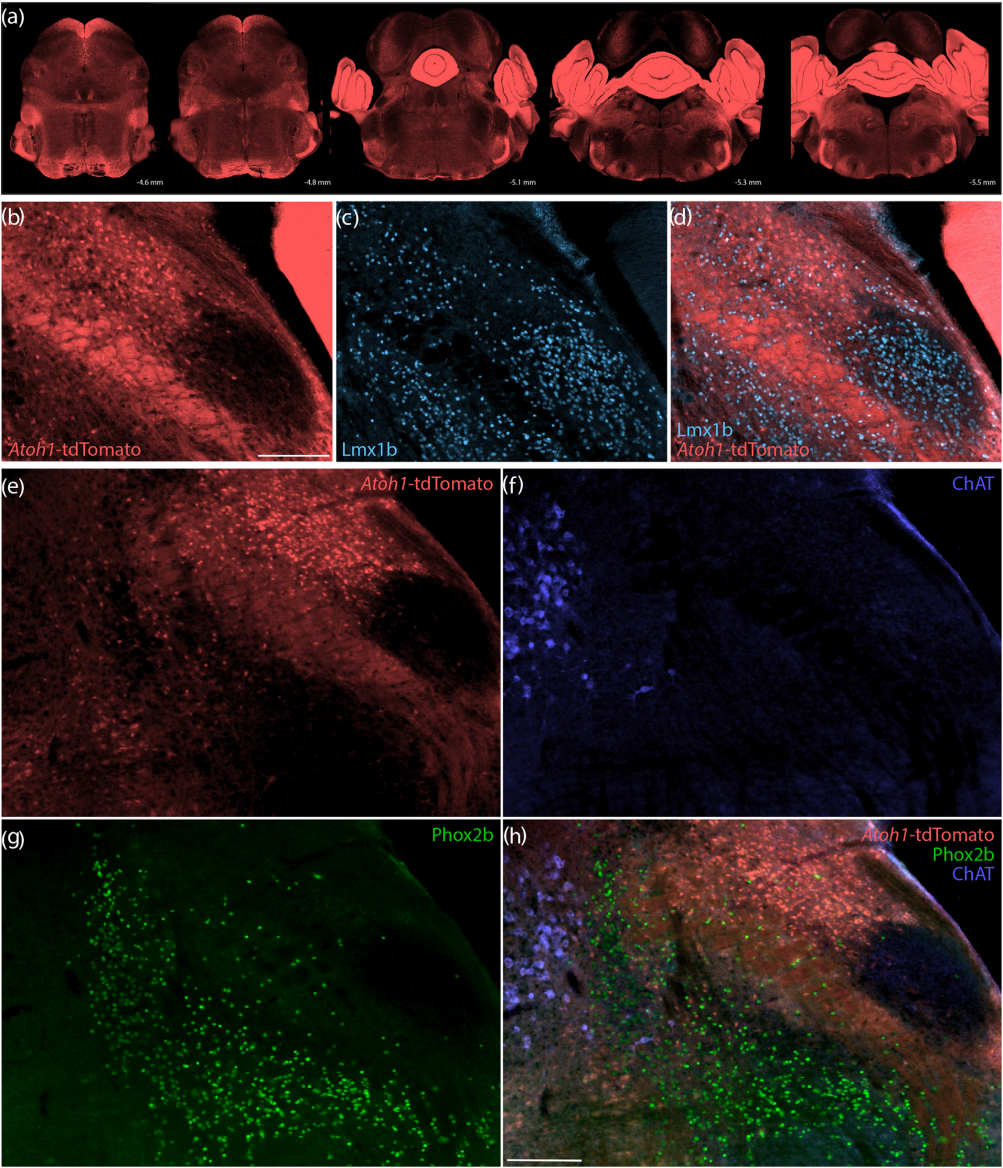
*Atoh1* Cre fate‐mapping with tdTomato. Cre fate‐mapping for *Atoh1* identified neurons in the cerebellum, PB, and several other brainstem regions, plus extensive labeling in fibrous processes. (a) Fluorescent reporter expression for *Atoh1‐Cre* (tdTomato, pseudocolored coral‐red) across five rostral‐to‐caudal tissue sections spanning the PB region. At each level, the approximate distance caudal to bregma is shown at bottom‐right. (b–d) Cre fate‐mapping for *Atoh1* followed by immunofluorescence labeling for Lmx1b (ice blue). (e–h) Immunofluorescence labeling for Phox2b (green) and ChAT (blue) at a mid‐level through the PB region (approximately bregma −5.1 mm). All scale bars are 200 μm. Scale bar in (b) also applies to panels (c, d). Scale bar in (h) also applies to panels (e–g)

Adult cells do not express *Atoh1*, so we used Cre fate‐mapping to identify *Atoh1*‐derived neurons. *Atoh1‐Cre;R26‐LSL‐tdTomato* mice had extensive tdTomato expression in the cerebellum and brainstem (*n* = 4, Figure 14a), similar to previous descriptions in other Cre‐reporter strains (Machold & Fishell, 2005; Wang et al., 2005). We found ubiquitous tdTomato expression in cerebellar granule cells, and none in Purkinje cells. Fibrous labeling pervaded the molecular layer, arbor vitae, superior cerebellar peduncle, and middle cerebellar peduncle. Outside the cerebellum, we found fibrous labeling in a ventral majority of the trigeminal principal sensory nucleus, which contrasted a near‐absence of labeling dorsally in this nucleus. The trigeminal motor nucleus and a round, central portion of the facial motor nucleus were both devoid of labeling, but the absence of labeling in these small regions contrasted a broader meshwork of labeled fibers in the surrounding brainstem. In the LC, tdTomato did not colocalize with tyrosine hydroxylase, but we found smaller, tdTomato‐expressing neurons scattered through the dorsal LC, and Barrington's nucleus contained large, tdTomato‐expressing neurons. We also identified a small number of tdTomato‐expressing neurons that contained Phox2b caudal and medial to the caudal LC, near the medial vestibular nucleus (not shown).

The PB contained many tdTomato‐expressing neurons, consistent with several previous reports (Machold & Fishell, 2005; Rose et al., 2009; van der Heijden & Zoghbi, 2018; Wang et al., 2005). The distribution of these putatively *Atoh1*‐derived neurons skewed dorsally, rostrally, and laterally, and many appeared to contain FoxP2. In contrast, tdTomato was largely absent from the ventrolateral PB (PBeL) and supratrigeminal region and did not appear to colocalize with either Lmx1b (Figure 14b–d) or Phox2b (Figure 14e–h). At mid‐levels of the PB, a dorsal cluster of tightly packed, larger *Atoh1*‐derived neurons resembled the rat “internal lateral” subnucleus (Bester et al., 1999; Bourgeais et al., 2001; Feil & Herbert, 1995; Fulwiler & Saper, 1984; Krout & Loewy, 2000). These neurons appeared to lack FoxP2, except at the ventral rim of this cluster. Besides these, we did not find any discrete clusters of tdTomato‐expressing neurons lacking FoxP2. The remainder of the lateral PB contained intermingled populations of *Atoh1*‐derived neurons with and without FoxP2 across a continuum of regions resembling the “dorsal lateral,” “central lateral,” and “superior lateral” subnuclei in rats (Fulwiler & Saper, 1984), plus a rostral‐ventral region we referred to as the “rostral‐to‐external‐lateral” subnucleus in mice (Geerling et al., 2016).

**FIGURE 15:**
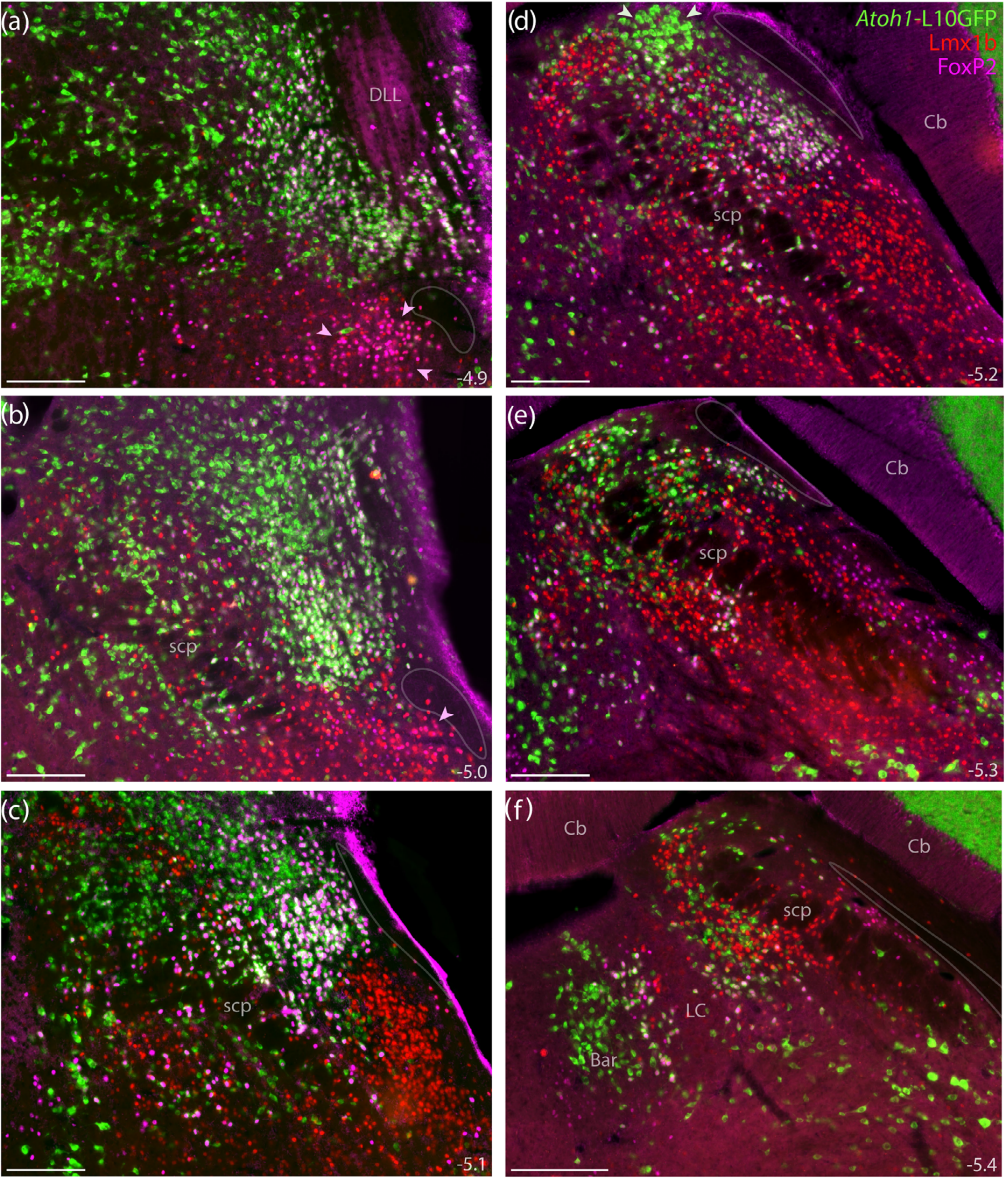
*Atoh1* Cre fate‐mapping with L10GFP. The L10GFP Cre‐reporter identified *Atoh1*‐derived cell bodies. Panels (a–f) show L10GFP expression across six rostral‐to‐caudal levels of the PB region in an *Atoh1‐Cre;R26‐lsl‐L10GFP* mouse after immunofluorescence labeling for Lmx1b (red) and FoxP2 (magenta). Nuclear FoxP2 immunofluorescence overlapping cytoplasmic L10GFP appears white. Arrowheads in panels (a, b) highlight the KF region. Arrowheads in (d) highlight a prominent, dorsal cluster of L10GFP‐expressing neurons. Translucent outlines in each panel show the location of a small white matter tract alongside the lateral PB and KF region, which is labeled “ventral spinocerebellar tract” in current brain atlases. Approximate distance caudal to bregma is provided at the bottom‐right of each panel (in mm). All scale bars are 200 μm

**FIGURE 16:**
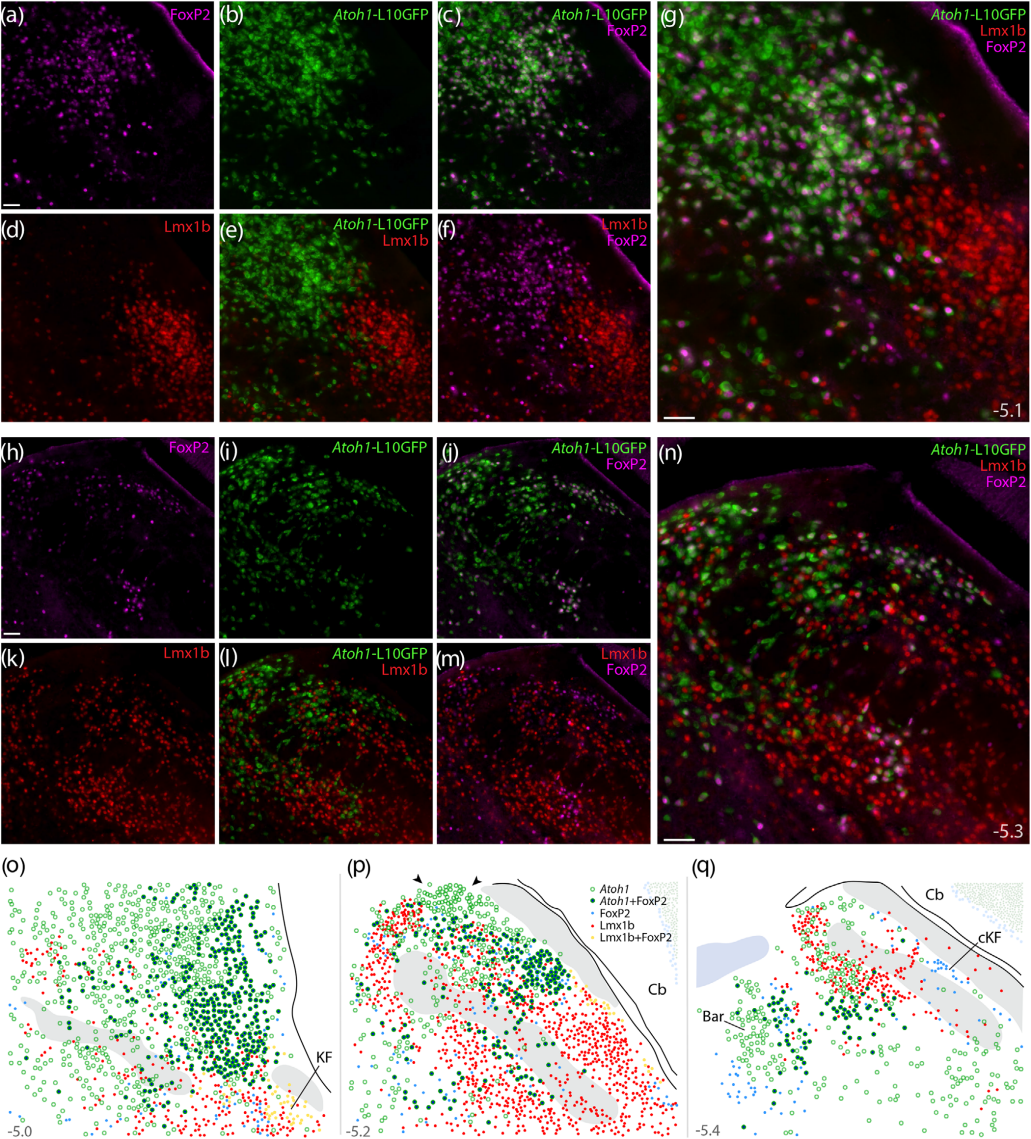
*Atoh1* Cre fate‐mapping with L10GFP: magnified color separations and plots. Panels (a–g) show immunofluorescence labeling for FoxP2 (magenta) and Lmx1b (red) after fate‐mapping for *Atoh1‐Cre* at a mid‐rostral level of the lateral PB. (h–n) Immunofluorescence labeling for FoxP2 and Lmx1b after L10GFP fate‐mapping for *Atoh1‐Cre* at a mid‐caudal level of the PB, centered over the “head” and “waist” of the scp. Approximate bregma levels are shown at bottom‐right in (g, n). All scale bars are 50 μm. Scale bar in (a) applies to (b–f) and scale bar in (h) applies to (i–m). (o, p) Rostral‐to‐caudal plots show the distribution of *Atoh1*‐derived neurons across the PB region, including large subsets with and without FoxP2. Arrowheads in (p) highlight a dorsal cluster of L10GFP‐expressing neurons that lack FoxP2. Throughout the PB, Lmx1b and L10GFP were mutually exclusive (no L10GFP‐expressing PB neurons contained Lmx1b). Approximate bregma levels are shown at bottom‐right in (g, n). Abbreviation: cKF, “caudal KF” population

**FIGURE 17:**
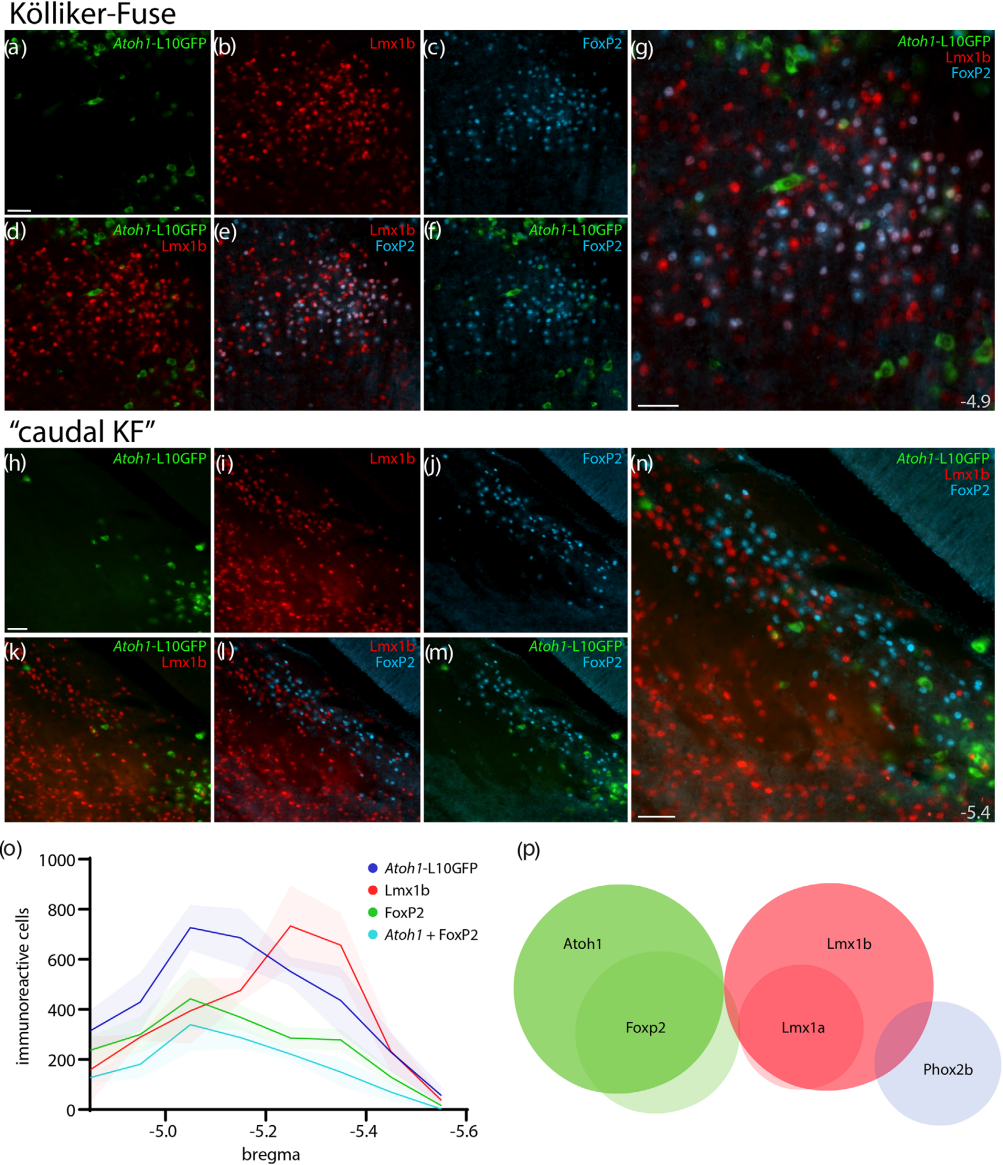
Cre fate‐mapping for *Atoh1* in KF and “caudal KF” with counts of *Atoh1*‐derived neurons across the PB region. Immunofluorescence labeling for Lmx1b (red) and FoxP2 (blue) after Cre fate‐mapping for *Atoh1* (green) in the KF region (a–g), and in the “caudal KF” (h–n). Approximate distance from bregma is shown at bottom‐right in (g, n). All scale bars are 50 μm. Scale bar in (a) also applies to (b–f). Scale bar in (h) also applies to (i–m). (o) Counts of *Atoh1*‐derived, FoxP2, Lmx1b, and *Atoh1*+FoxP2 double‐labeled neurons across six rostro‐caudal levels. Counts were averaged at each rostrocaudal level (*n* = 3 mice), with variance represented by a standard deviation envelope. Approximate distance caudal to bregma is shown on the *x*‐axis. (p) Venn diagram of transcription factor markers that identify neuronal populations in the PB region

The dense, fibrous background of axonal and dendritic tdTomato made it difficult to distinguish individual neurons and colocalize other molecular markers (Figure 14), so we crossed *Atoh1‐Cre* mice to the L10GFP Cre‐reporter strain described above. In the resulting progeny (*Atoh1‐Cre;R26‐lsl‐L10GFP*, *n* = 6), the GFP reporter concentrated in cell bodies, rather than axons and dendrites, allowing us to more definitively distinguish *Atoh1*‐derived neurons and colocalize molecular markers (Figures 15, 16, 17).

Within the PB, we did not find any cells containing both L10GFP and Lmx1b, indicating total mutual exclusivity between *Atoh1*‐derived neurons and *Lmx1b* expression. At the dorsal edge of the principal sensory trigeminal nucleus, we occasionally found an L10GFP‐expressing neuron with an Lmx1b‐immunoreactive nucleus, but these rare cells represented just 0.002% of the 12,606 L10GFP‐expressing neurons counted across this region (*n* = 3 mice). We also found mutual exclusivity between L10GFP and Phox2b in the PB and LC, although sparse *Atoh1*‐derived neurons located caudal and ventral to the LC did contain Phox2b along the medial vestibular nucleus and facial nerve genu.

In contrast to their mutual exclusivity with Lmx1b, many *Atoh1*‐derived PB neurons contained FoxP2. As in the tdTomato strain, double‐labeled neurons intermingled with other *Atoh1*‐derived PB neurons that lacked FoxP2 (Figures 15 and 16). At caudal levels, these intermingled populations (*Atoh1*‐derived neurons with and without FoxP2) extended around and through the superior cerebellar peduncle and intermingled extensively with Lmx1b‐immunoreactive neurons (Figure 15e,f). At middle and rostral levels, *Atoh1*‐derived neurons filled the lateral PB except for PBeL (Figure 15a–d). At rostral levels, their distribution extended up to the dorsal nucleus of the lateral lemniscus and down to the PB‐KF border (Figure 15a).

At mid‐levels of the PB, we again found a prominent, dorsal cluster of *Atoh1*‐derived neurons resembling the rat “internal lateral” subnucleus (arrowheads in Figures 15d and 16p). These neurons lacked FoxP2 and bordered a thin white matter tract that is labeled “ventral spinocerebellar tract” in current atlases (Dong, 2008; Paxinos & Franklin, 2013). Outside this one cluster, *Atoh1*‐derived neurons with and without FoxP2 intermingled extensively. FoxP2 colocalization was most prevalent near PBeL (Figures 15b–d and 16a–g), less prevalent dorsally, and least prevalent dorsomedially, near the head and waist of the superior cerebellar peduncle (Figure 16h–n). Also intermingling with these subsets were fewer neurons containing FoxP2 without L10GFP; these neurons did not form any discrete clusters except for the “caudal KF” population ventrolateral to the PB.

At rostral levels, none of the KF populations described above (neurons containing Lmx1b, Lmx1b+FoxP2, Lmx1b+Phox2b, or Phox2b) expressed L10GFP. Nor did we find L10GFP expression in the contiguous “lateral crescent” neurons alongside PBeL. Also, L10GFP was not expressed in any “caudal KF” neurons (Figure 17). Thus, we did not find any evidence to support previous claims that KF neurons derive from *Atoh1*‐expressing precursors in the rhombic lip (Gray, 2008; van der Heijden & Zoghbi, 2018).

### 3.9 Interim summary

Identifying the large population of *Atoh1*‐derived PB neurons that lack FoxP2 completed a core set of developmental‐genetic markers useful for classifying neurons in this region. Specifically, *Lmx1* paralogs (primarily *Lmx1b*, but also *Lmx1a*) and *Atoh1* define two, mutually exclusive macropopulations of glutamatergic neurons (Figure 17p). We designed our experiments to assess colocalization, rather than total numbers, but as a rough estimate, summing these two macropopulations suggests that there are approximately 20,000 PB neurons on each side of the mouse brainstem (21,472 ± 1636; *n* = 3 mice).

### 3.10 Subpopulations of Lmx1‐ and Atoh1‐derived PB neurons

Transcription factors influence the repertoire of genes (including other transcription factors) that neurons transcribe, and this “transcriptome” shapes the pattern of synapses a neuron establishes with other neurons (Hirsch et al., 2021). Therefore, using transcription factors to classify PB neurons should help us interpret other patterns of gene expression and neural circuit connectivity. For example, under a cytoarchitectural framework, *Foxp2* expression marked an indiscrete population of similar‐appearing cells, strewn across virtually every PB subnucleus (Figure 2). In contrast, our new framework identifies two glutamatergic subpopulations (a large subset of *Atoh1*‐derived PB neurons and a small subset of *Lmx1*‐derived neurons; Figure 19) and a GABAergic subpopulation (“caudal KF”). Our molecular framework also revealed novel, diverse subsets of KF neurons (Phox2b‐only, Phox2b+Lmx1b, FoxP2+Lmx1b, Lmx1b‐only; Figure 10). Cataloging all the genes that distinguish subsidiary PB subpopulations is not within the scope of this study, where our goal is to provide a molecular framework for interpreting such data. Nonetheless, to show the predictive advantages of a molecular ontology, we will highlight two further subpopulations within each PB macropopulation.

**FIGURE 18:**
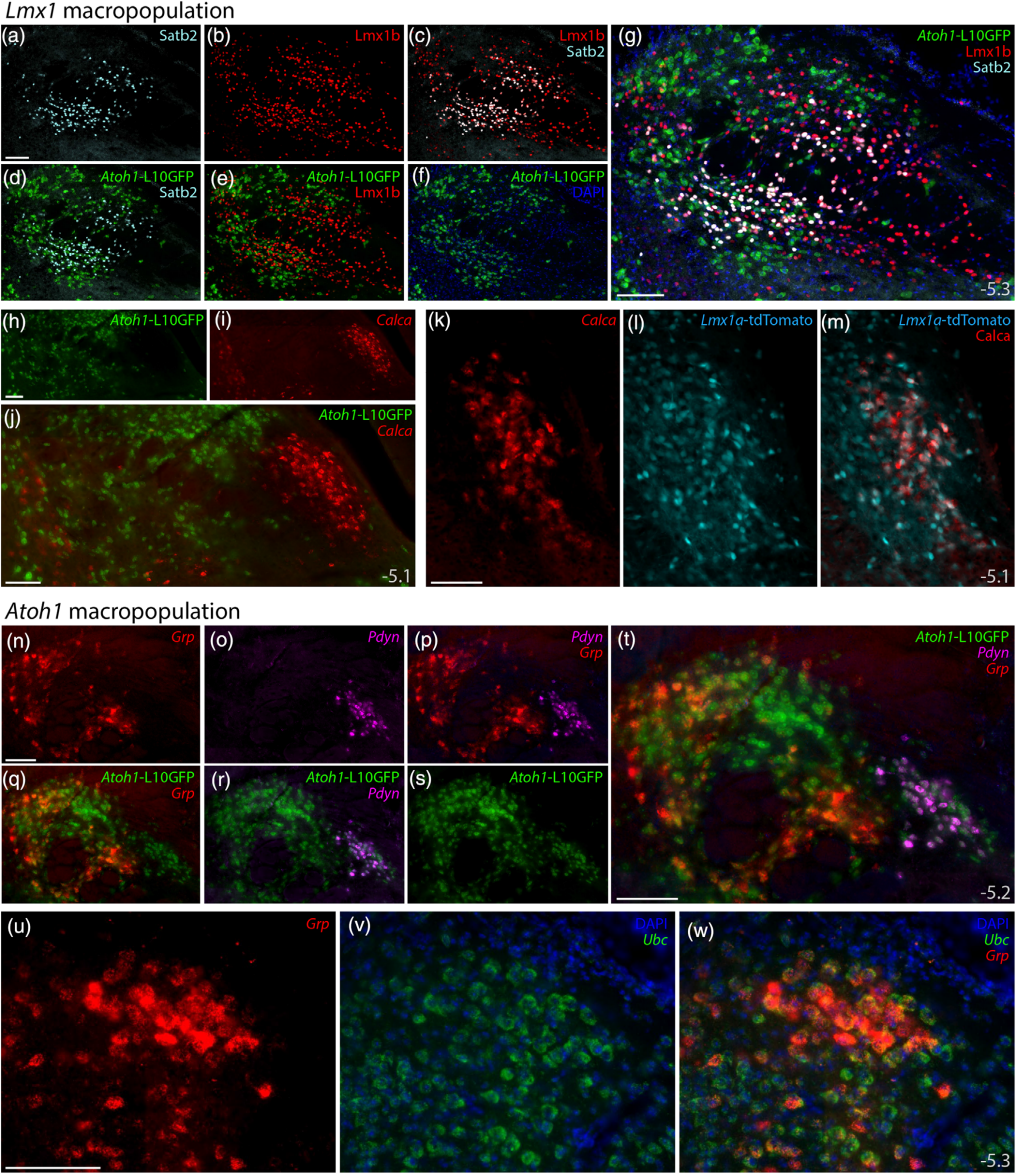
Subpopulations of *Lmx1*‐ and *Atoh1*‐derived PB neurons. Subsidiary genes define separate subpopulations within the *Atoh1*‐ and *Lmx1*‐derived PB macropopulations. (a–g) Immunofluorescence labeling for Satb2 (light blue) and Lmx1b (red) with L10GFP Cre‐reporter for *Atoh1* (green) at a mid‐caudal level of the PB. (h–j) FISH labeling for *Calca* (red) with L10GFP Cre‐reporter for *Atoh1* (green) at a mid‐level of the PB. (k–m) FISH labeling for *Calca* with tdTomato (pseudocolored ice blue) Cre‐reporter for *Lmx1a* in PBeL. (n–t) FISH labeling for *Grp* mRNA (red) and *Pdyn* mRNA (magenta) with L10GFP Cre‐reporter for *Atoh1* (green) at a mid‐caudal level of the PB. (u–w) FISH labeling for *Grp* mRNA (red) in a dorsal cluster of larger neurons that resemble the “internal lateral” subnucleus of the rat PB (Fulwiler & Saper, 1984), with *Ubc* mRNA (green) shown for neuroanatomical background. Approximate bregma levels shown at bottom‐right in (g, j, m, t, w). All scale bars are 100 μm and apply to similar panels

**FIGURE 19:**
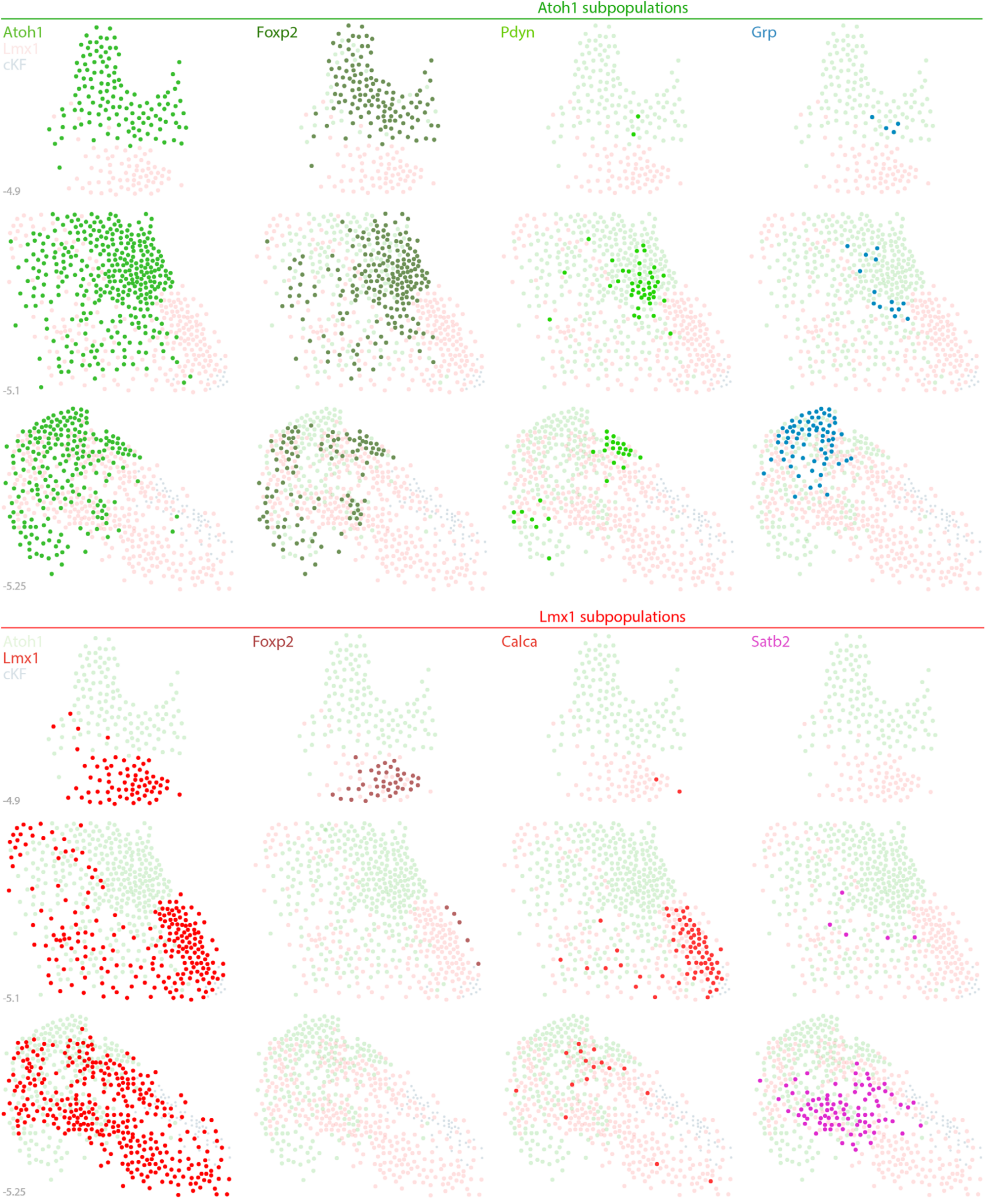
Distribution of *Atoh1* and *Lmx1* subpopulations. The PB contains two neuronal macropopulations, which derive from embryonic precursors expressing *Atoh1* (green) or *Lmx1* (*Lmx1b* and *Lmx1a*; red). This figure illustrates the rostral‐to‐caudal distribution of subsidiary populations within each macropopulation. Some genetic markers highlight a subset of just one macropopulation, exemplified by *Pdyn* and *Grp* (*Atoh1* macropopulation) and by *Calca* and *Satb2* (*Lmx1* macropopulation). In contrast, *Foxp2* identifies separate subsets within each macropopulation. *Foxp2* is expressed by an extensive subset of *Atoh1*‐derived neurons (dark‐green dots) and by a more restricted, ventral subset of the *Lmx1* macropopulation that is located rostrally, in the KF (dark‐red dots), separate from the caudal population of GABAergic FoxP2 neurons in the “caudal KF” (cKF, light blue dots). Approximate level caudal to bregma (in mm) is shown at bottom‐left for each of the three PB levels illustrated

First, the transcription factor *Satb2* marks PB neurons that relay gustatory information to the thalamus (Fu et al., 2019; Huang et al., 2013; Jarvie et al., 2021; Maeda et al., 2009). We confirmed that both *Satb2* mRNA (not shown) and Satb2 immunofluorescence (Figure 18a) identify a restricted distribution of PB neurons, primarily at caudal levels. *Satb2*‐expressing neurons distributed within and near fascicles of the superior cerebellar peduncle, spanning subregions homologous to the “medial,” “waist,” “ventral lateral,” and “inner PBeL” subnuclei in rats (Fulwiler & Saper, 1984). Satb2 and FoxP2 did not colocalize (not shown), nor did we find Satb2 in any *Atoh1*‐derived neurons (Figure 18d). Instead, every Satb2‐labeled neuron contained Lmx1b (Figure 18c,g). Therefore, gustatory relay neurons expressing *Satb2* are a subset of the *Lmx1* PB macropopulation (Figure 19).

Next, we examined the expression of *Calca*, which encodes the neuropeptide CGRP (calcitonin gene‐related peptide). *Calca*‐expressing PB neurons form a critical link in signaling pathways for conditioned taste aversion, malaise, and anorexia, plus alerting responses to aversive stimuli that include pain, itch, and hypercarbia (Carter et al., 2015; Chen et al., 2018; Kaur et al., 2017; Palmiter, 2018; Saper, 2016). We found *Calca* mRNA in the outer part of PBeL and in other neurons described previously (Huang et al., 2021), but not in any *Atoh1*‐derived neurons (Figure 18h–j), consistent with the mutual exclusivity between CGRP and FoxP2 in this region (Huang et al., 2021). Instead, labeling *Calca* mRNA in *Lmx1a* Cre‐reporter mice revealed extensive colocalization with tdTomato (Figure 18k–m), resembling the colocalization between CGRP and Lmx1b in both rats and mice (Huang et al., 2021; Miller et al., 2012). Therefore, like *Satb2*, *Calca* expression identifies a subset of the *Lmx1* PB macropopulation (Figure 19).

We then identified two markers within the *Atoh1* macropopulation. Labeling *Pdyn* mRNA, which encodes the neuropeptide dynorphin, identified a distribution resembling previous reports in rats and mice (Geerling et al., 2016; Hermanson et al., 1998; Huang et al., 2020; Miller et al., 2012). *Pdyn* expression in the mouse PB identified neurons in regions resembling the “dorsal lateral” (Figure 18r) and “central lateral” subnuclei in rats (Fulwiler & Saper, 1984), plus a caudal subset of *Pdyn*‐expressing neurons in the “pre‐LC” population that spans the medial PB and dorsal LC (see Gasparini et al., 2021), and all these neurons expressed L10GFP. Therefore, *Pdyn*‐expressing PB neurons are a subset of the larger *Atoh1* macropopulation (Figure 19).

Another neuropeptide, gastrin‐releasing peptide (*Grp*), identified a separate subpopulation (Figure 18q). At mid‐caudal levels, *Grp*‐expressing neurons occupied a dorsomedial subregion, as reported in rats (Figure 6H of Wada et al., 1990), surrounding the “head” and “waist” of the superior cerebellar peduncle (Figure 18p). At mid‐levels of the PB, *Grp* identified a prominent, dorsal cluster (Figure 18u–w) resembling the rat “internal lateral” subnucleus (Fulwiler & Saper, 1984). Further rostrally, *Grp*‐expressing neurons concentrated near PBeL and expressed *Foxp2* but not *Calca* (not shown). Overall, *Grp* expression identifies another, distinct subset within the *Atoh1* PB macropopulation (Figure 19).

### 3.11 Genetic segregation of axonal trajectories and output targets

Embryonic transcription factors establish patterns of neurogenesis and axon growth needed for adult circuit functions. Connectivity patterns that define and distinguish adult neurons often reflect their separate developmental lineages, which are determined in large part by embryonic transcription factors (Hirsch et al., 2021). Thus, we hypothesized that macropopulation identity determines the trajectory and targets of axons projecting from the PB to distal brain regions.

**FIGURE 20:**
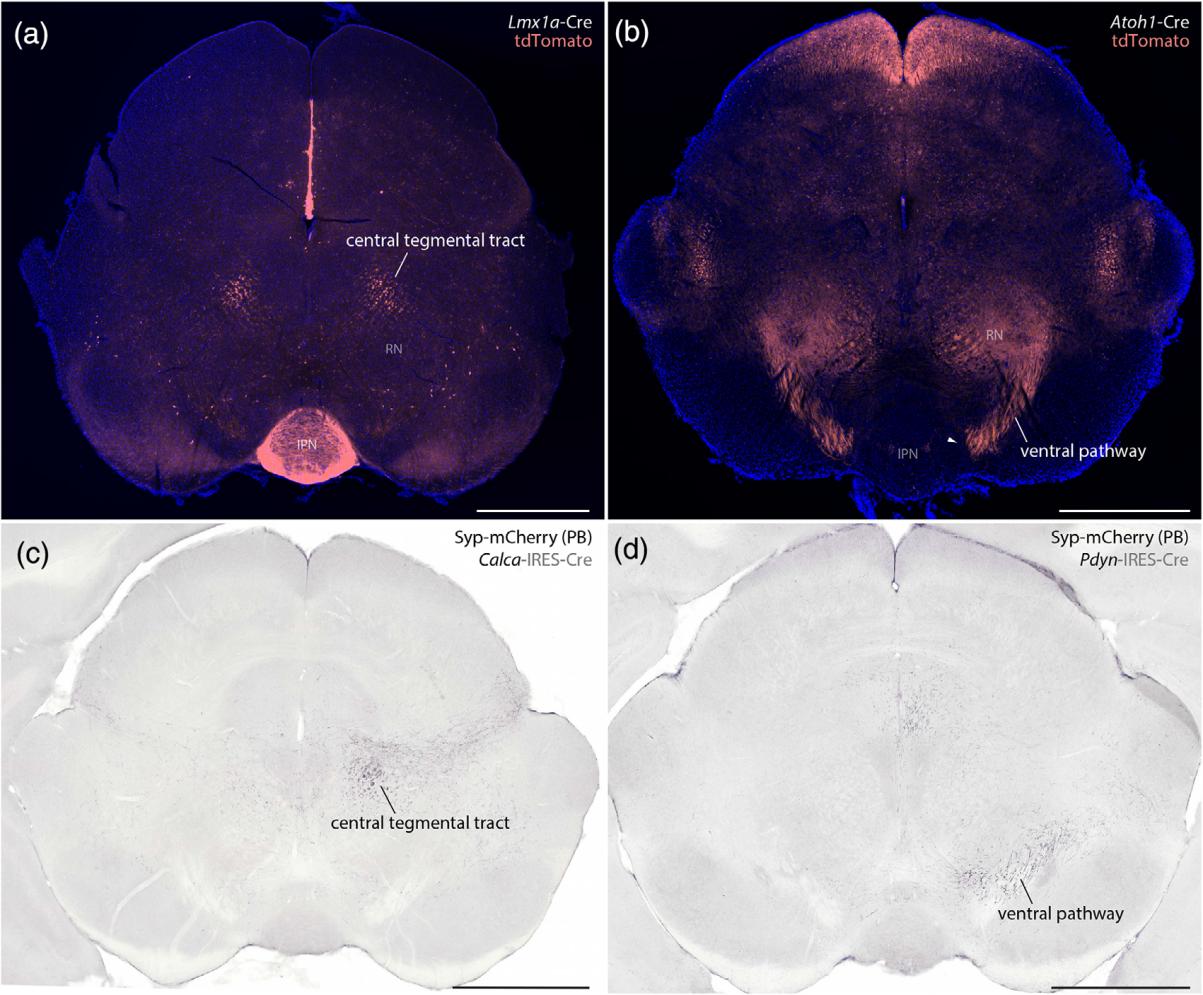
Axonal trajectories of *Lmx1*‐ and *Atoh1*‐derived PB neurons. (a) *Lmx1a* Cre‐reporter labeling in the midbrain (tdTomato, pseudocolored coral‐red). (b) *Atoh1* Cre‐reporter labeling in the midbrain (tdTomato, pseudocolored coral‐red). (c) Nickel‐DAB immunolabeling for synaptophysin‐mCherry (Syp‐mCherry) in the midbrain of a *Calca‐Cre* mouse after injection of AAV‐DIO‐Syp‐mCherry into the PB. (d) Nickel‐DAB immunolabeling for Syp‐mCherry in the midbrain of a *Pdyn*‐IRES‐Cre mouse after injection of AAV‐DIO‐Syp‐mCherry into the PB. Abbreviations: IPN, interpeduncular nucleus; RN, red nucleus. All scale bars are 1 mm

First, to test whether developmental‐genetic identity predicts axonal trajectory, we compared axonal labeling in the midbrain, rostral to the PB, after Cre fate‐mapping with tdTomato. In *Lmx1a* Cre‐reporter mice, we found tdTomato labeling in fascicles of axons within the central tegmental tract (CTT), both in late‐stage embryos (E17.5, *n* = 2; not shown) and in adult mice (*n* = 2, Figure 20a). In every case, this fibrous tdTomato labeling in the CTT traced back to an origin in the PB (not shown). In contrast, the CTT was unlabeled in *Atoh1* Cre‐reporter mice (*n* = 4). In these mice, we instead found fibrous tdTomato labeling in the superior cerebellar peduncle (arborizing extensively in the red nucleus) and in the medial lemniscus (arrowhead in Figure 20b), plus a contiguous band of axons lateral to the medial lemniscus, in‐between the red nucleus and substantia nigra. *Lmx1a* Cre‐reporter mice did not have any axonal labeling in this ventral pathway.

These dichotomous developmental patterns matched patterns of axonal labeling we identified after transducing adult PB neurons that express *Calca* (*Lmx1* macropopulation) or *Pdyn* (*Atoh1* macropopulation). Our previous injections of AAV8‐DIO‐synaptophysin‐mCherry into the PB (Huang et al., 2020; Huang et al., 2021) produced axonal labeling in either the CTT (*Calca* cases; Figure 20c) or ventral pathway (*Pdyn* cases; Figure 20d). The strikingly similar results of developmental fate‐mapping and adult Cre‐conditional labeling support our hypothesis that macropopulation identity predicts the trajectory of PB axonal projections. Specifically, neurons in the *Lmx1* PB macropopulation extend axons through the CTT (between the oculomotor nucleus and red nucleus), while neurons in the *Atoh1* PB macropopulation extend axons through a ventral pathway (between the red nucleus and substantia nigra, alongside the medial lemniscus).

Next, to test whether developmental‐genetic identity also predicts the distal targets of these axonal projections, we combined Cre fate‐mapping with retrograde axonal tracing. In *Atoh1‐Cre*;*R26‐lsl‐L10GFP* mice, we injected a retrograde tracer (cholera toxin b subunit, CTb) into brain regions that receive heavy input from glutamatergic PB neurons (Huang et al., 2020). We then compared L10GFP expression (marking *Atoh1*‐derived neurons) with immunolabeling for Lmx1b and CTb.

**FIGURE 21:**
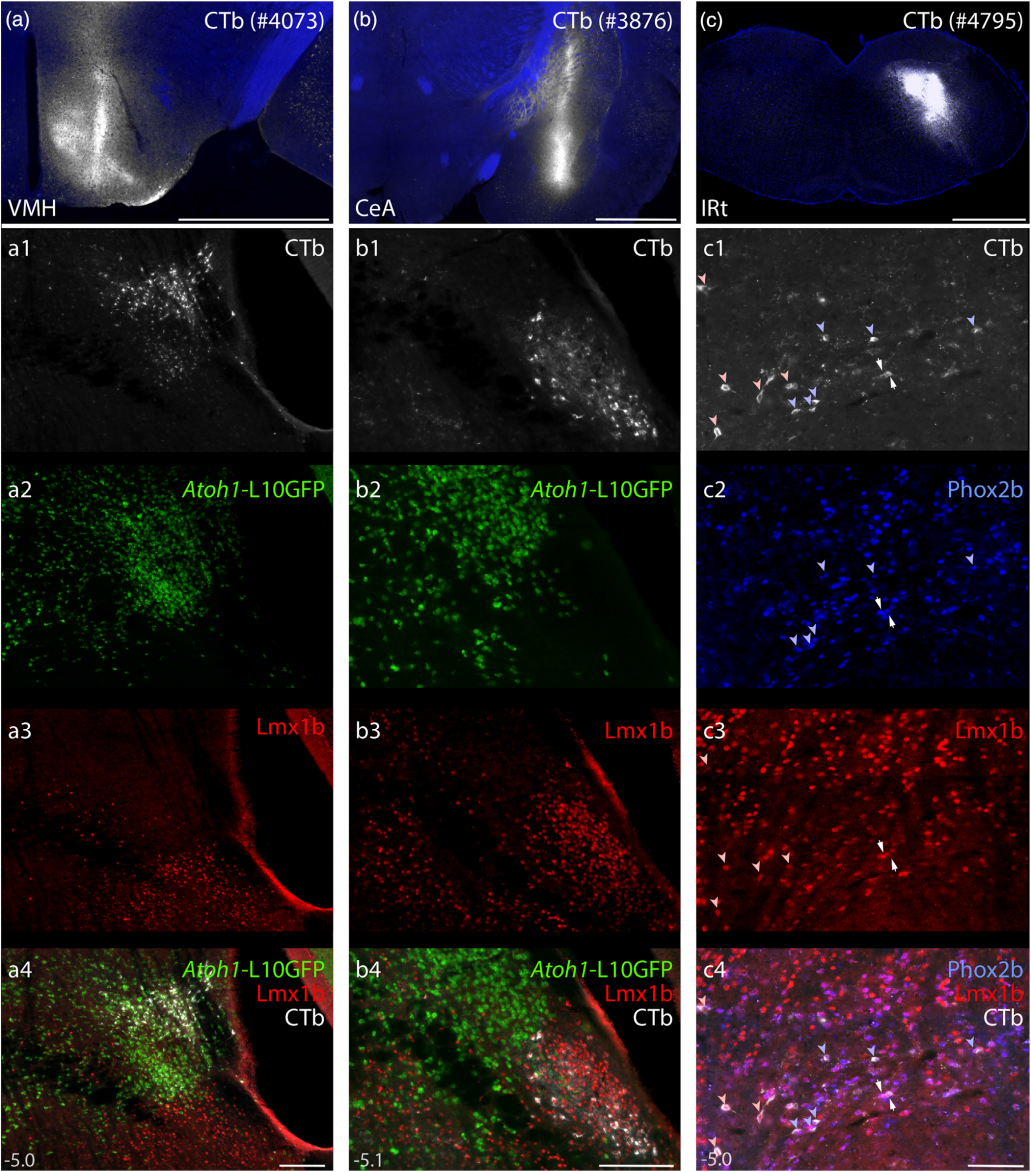
Distal targets of axonal projections from separate PB macropopulations. Neurons in the PB region contained retrograde CTb labeling after tracer injections into the ventromedial hypothalamic nucleus (VMH), central nucleus of the amygdala (CeA), and intermediate reticular formation (IRt). (a–c) CTb injection sites (white). (a1–a4) Immunofluorescence labeling for CTb (white) after tracer injection into the VMH along with L10GFP Cre‐reporter expression in *Atoh1*‐derived neurons and Lmx1b immunofluorescence (red) at a level approximately 5.0 mm caudal to bregma. (b1–b4) Immunofluorescence labeling for CTb after tracer injection into the CeA, along with L10GFP Cre‐reporter for *Atoh1* and Lmx1b immunofluorescence (approximately 5.1 mm caudal to bregma). (c1–c4) Immunofluorescence labeling for CTb, Phox2b (blue), and Lmx1b along the ventral border of the PB. Light blue arrowheads indicate neurons that contain both CTb and Phox2b. Light red arrowheads indicate neurons that contain both CTb and Lmx1b. White arrows highlight neurons that contain CTb and both transcription factors. Approximate bregma levels are shown at bottom‐left in (a4, b4, c4). Scale bars in (a–c) are 1 mm. Scale bars in (a4–c4) are 200 μm and apply to panels above

**FIGURE 22:**
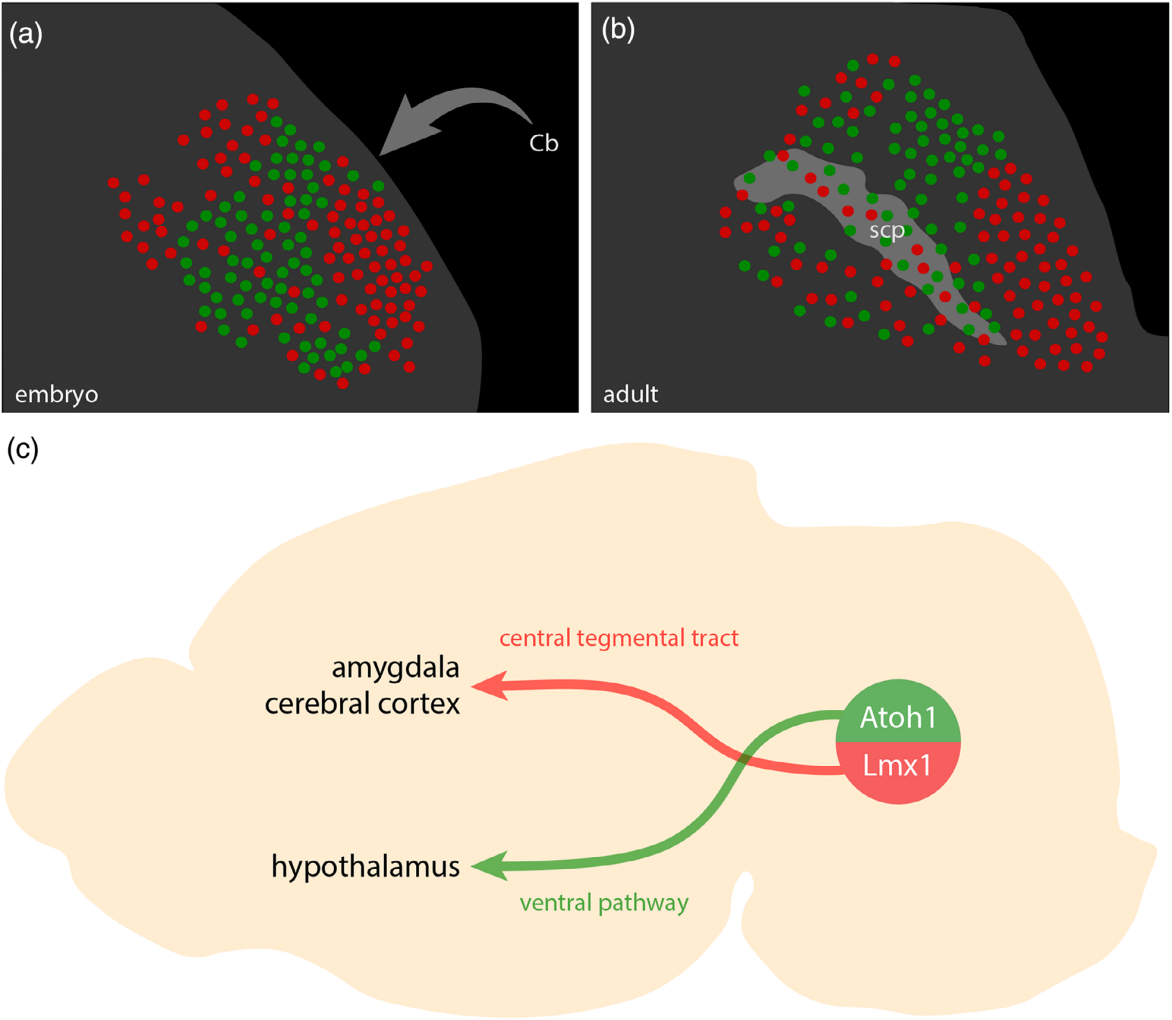
Summary. (a, b) Illustrated distribution of *Atoh1* (green) and *Lmx1* (red) PB neurons in late‐embryonic (a) and adult (b) mice. Cerebellar axons project through this region and form the scp, dispersing PB neurons. (c) PB neurons in separate *Atoh1* and *Lmx1* macropopulations project axons through separate pathways to separate forebrain targets. Note that neurons in each macropopulation project to additional brain regions (see Huang et al., 2021). Hindbrain projections from ventral populations are not shown

After injections in the ventromedial hypothalamus (*n* = 6), retrogradely labeled neurons expressed L10GFP. Most of these neurons concentrated rostrally, in a dorsolateral subregion of the PB (Figure 21a), but their distribution also extended caudally within the lateral PB (not shown). After injections in the central nucleus of the amygdala (*n* = 2) or insular cortex (*n* = 1; not shown), retrogradely labeled PB neurons lacked L10GFP and instead contained Lmx1b. Many of these neurons concentrated in PBeL (Figure 21b), and their distribution extended caudally, across the superior cerebellar peduncle (not shown). These retrograde tracing results complement the anterograde labeling patterns we reported after transducing PB neurons that express either *Calca* or *Pdyn* (Huang et al., 2021) and support our hypothesis that neurons in separate *Lmx1* and *Atoh1* PB macropopulations project axons to separate target sites in the forebrain (Figure 22c). Of note, these findings do not imply that all neurons in the *Atoh1* macropopulation project axons to the hypothalamus, or that all neurons in the *Lmx1* macropopulation project axons to the amygdala, and further experiments are needed to determine the full array of brain regions targeted by neurons within each macropopulation.

Ventral to the PB, we predicted that neurons expressing *Phox2b* and *Lmx1b* project axons to the hindbrain. Many neurons in this supratrigeminal region receive proprioceptive input from the trigeminal nerve and send output to cranial motor neurons and hindbrain interneurons (Li et al., 2005; Marfurt & Rajchert, 1991; Rokx et al., 1986; Stanek et al., 2016), and we found that glutamatergic neurons here project axons to the hindbrain reticular formation (Geerling et al., 2017; Huang et al., 2020). We also confirmed in mice that injecting CTb into the medullary reticular formation produces retrograde labeling in neurons along the ventral edge of the rostral PB (Huang et al., 2021). To test whether these neurons contain Phox2b or Lmx1b, we made additional CTb injections into the medullary reticular formation (*n* = 4; Figure 21c), then immunolabeled Phox2b, Lmx1b, and CTb. In these cases, CTb‐labeled neurons distributed along the ventral PB border. Many contained Phox2b (with or without Lmx1b), and some contained Lmx1b without Phox2b (Figure 21c 1–c4). Therefore, glutamatergic neurons expressing *Phox2b* or *Lmx1b* immediately ventral to the PB project axons to the hindbrain reticular formation.

## 4 DISCUSSION

Our findings lay the foundation for a new ontology that uses intrinsic, molecular information to identify PB neurons. This molecular ontology offers an observer‐independent alternative to taxonomies that are based on the position of a white matter tract that disperses PB neurons (Figure 22a,b). Molecular features blur some of the atlas boundaries inferred from Nissl cytoarchitecture and reveal an intermingled web of developmentally distinct macropopulations spanning the superior cerebellar peduncle, challenging its utility for subdividing the PB. Beyond laying the groundwork for a new way to classify PB neurons, the information presented here opens opportunities to improve our understanding of interoceptive neural circuit functions.

### 4.1 Practical implications, limitations, and opportunities

Our findings represent the first comprehensive framework for interpreting neuron‐type‐specific molecular information in the PB region. Adopting this developmental‐genetic framework will help investigators organize transcriptomic data and focus future attempts to identify and study PB neurons.

Transcriptomic data from an adult brainstem should be sufficient to classify glutamatergic neurons that express *Lmx1a/b* and other adult markers, but adult neurons do not express *Atoh1* (Machold et al., 2011), so identifying *Atoh1*‐derived subpopulations that do not express *Foxp2* may require a fate‐mapping strategy like the one used here. Among these *Atoh1*‐derived PB neurons that do not express *Foxp2*, we identified a large, *Grp*‐expressing subset. This *Grp* subset likely contains further subpopulations, prominently including a dorsal cluster resembling the “internal lateral” subnucleus in rats (Fulwiler & Saper, 1984; Paxinos & Watson, 2007). Neurons in this cluster relay pain‐related information from the spinal cord to the thalamus (Bernard et al., 1995; Bester et al., 1999; Bester et al., 1995; Bourgeais et al., 2001; Feil & Herbert, 1995; Kitamura et al., 1993; Krout & Loewy, 2000), but the term “internal lateral” was omitted from a major mouse brain atlas (Dong, 2008; Wang et al., 2020) and its location was added to a separate subnucleus (“superior lateral”), which had been distinguished in rats as sending axonal projections to the hypothalamus (Bester et al., 1997; Bester et al., 1995; Fulwiler & Saper, 1984). This new taxonomy––substituting “superior lateral” for “internal lateral” in mice––appeared in subsequent studies of neurons that relay noxious spinoparabrachial information to the thalamus (Barik et al., 2021; Deng et al., 2020). In contrast to many other findings that blur atlas boundaries, our results unambiguously support the cytoarchitectonic distinction of an “internal lateral” population (Fulwiler & Saper, 1984) by identifying these neurons as a *Foxp2*‐negative subset of the *Atoh1* macropopulation. Except for a rostral subset that express *Foxp2*, *Grp*‐expressing neurons resemble the distribution of PB neurons that express the substance P receptor *Tacr1* (Barik et al., 2021), which fail to develop in mice with *Atoh1* deletion from the rostral rhombic lip (van der Heijden & Zoghbi, 2018). The *Grp* distribution also partly resembles the distribution of *Penk*‐expressing PB neurons, a subset of which project axons to the thalamus (Hermanson & Blomqvist, 1997). We are testing several additional markers (unpublished) and expect that single‐cell transcriptomic analysis will identify a larger suite of neuronal subpopulations within the *Atoh1* macropopulation.

From a purely practical perspective, it is useful to classify neurons using developmental‐genetic features, rather than cytoarchitecture, because the resulting information can guide genetically targeted experiments that evaluate hypotheses about specific subpopulations. Until recently, studying the PB involved inserting an electrode, needle, micropipette, or cannula (to stimulate, inhibit, destroy, trace, or record neurons) and then using a cytoarchitectural taxonomy to correlate experimental results with histological location in each brain. Most injection sites and implants were larger than most PB subpopulations, which intermingle extensively, so these approaches simultaneously targeted subsets of PB neurons with distinct connections and functions. Now, genetic methods allow investigators to target more specific subpopulations, including an overlapping potpourri of neurons that express *Satb2, Calca*, *Pdyn, Cck*, *Oxtr1*, *Htr2c*, *Oprm1*, or *Tacr1* (Barik et al., 2021; Chiang et al., 2020; Fu et al., 2019; Garfield et al., 2014; Jarvie et al., 2021; Kaur et al., 2017; Liu et al., 2021; Norris et al., 2021; Palmiter, 2018; Park et al., 2020; Ryan et al., 2017; Yang et al., 2020). Limiting the immense potential of this approach were the lack of a framework for understanding ontological relationships between subpopulations and a lack of markers for remaining PB neurons.

Now, learning that the PB contains two, genetically distinct macropopulations has immediate practical advantages. For example, investigators can rapidly focus the hunt for a subpopulation mediating a particular effect by separately ablating or stimulating each macropopulation to exclude one or the other half of all PB neurons. Even without a full and final menu of markers to distinguish every remaining subpopulation, combining our framework with existing information opens opportunities to access previously indistinguishable subsets by using intersectional genetic targeting methods (Branda & Dymecki, 2004; Fenno et al., 2014; Madisen et al., 2015; Weinholtz & Castle, 2021). As an example, retrograde axonal tracing identified two, distinct subsets of *Cck*‐expressing PB neurons: one sends output to the cerebral cortex, and the other to the hypothalamus (Grady et al., 2020). Using intersectional methods to target *Cck*‐expressing neurons derived from precursors expressing either *Lmx1b* (cortex‐projecting) or *Atoh1* (hypothalamus‐projecting) should provide the selective access needed to isolate and test each subset. Intersectional methods will be particularly important for studying KF neurons (discussed below).

### 4.2 Comparison with previous neurodevelopmental literature

Our study is the first to identify the PB as a blend of two, developmentally distinct macropopulations. Previous descriptions of *Lmx1a* and *Lmx1b* here did not mention *Atoh1* (Asbreuk et al., 2002; Dai et al., 2008; Miller et al., 2012; Sarropoulos et al., 2021; Zou et al., 2009), and initial studies reporting *Atoh1*‐derived PB neurons did not mention *Lmx1a* or *Lmx1b* (Machold & Fishell, 2005; Rose et al., 2009; van der Heijden & Zoghbi, 2018; Wang et al., 2005), leaving the ontological relationships between these populations unclear. One study reported that many PB neurons fail to develop in *Atoh1*‐null mice (Rose et al., 2009) and showed a loss of *Slc17a6* (Vglut2) mRNA at a far‐rostral level without showing middle or caudal levels of the PB or labeling markers that might have identified neurons in the *Lmx1* macropopulation. Another study reported that all *Atoh1*‐derived PB neurons contain CGRP (van der Heijden & Zoghbi, 2018), which is incompatible with the mutual exclusivity between *Calca* mRNA and *Atoh1*‐derived neurons (Figure 18h–j). This previous result likely involved off‐target immunoreactivity because there is little overlap between the distribution of *Atoh1*‐derived neurons and that of *Calca* mRNA, or of CGRP labeling produced by antisera validated in knockout mice (Huang et al., 2021).

In the embryonic (E14) brainstem, gene‐chip analysis identified *Foxp2* expression in *Atoh1*‐derived neurons (Machold et al., 2011). In neonatal (P0) mice, another study identified FoxP2 immunoreactivity in *Atoh1*‐derived neurons that appear to be located in the lateral PB but were labeled “KF” (Figures 3 and 4 of Gray, 2008). Other images from the same study showed Lmx1b immunoreactivity in what appears to be the external lateral PB (labeled “Pr5” in Figure 3E of Gray, 2008), plus a ventrolateral cluster of FoxP2‐immunoreactive, *Ptf1a*‐derived neurons resembling the “caudal KF” (Figure 4A of Gray, 2008). Besides discrepant nomenclature, these results are consistent with our conclusion that adult *Foxp2* expression identifies three, developmentally distinct subpopulations here: (1) a small, rostral subset of *Lmx1*‐derived glutamatergic neurons (within KF); (2) a large subset of *Atoh1*‐derived glutamatergic neurons (within PB); and (3) a small, ventrolateral cluster of *Ptf1a*‐derived GABAergic neurons (“caudal KF”).

### 4.3 Functional implications

Before genetic markers, several studies focused on PB neurons that we now identify as subsets within the *Lmx1* macropopulation. Early work on gustatory pathways (Norgren & Leonard, 1971, 1973) paved the way for the discovery that *Satb2*‐expressing PB neurons relay taste information (Fu et al., 2019; Jarvie et al., 2021). Similarly, early work on PB neurons that relay viscerosensory information to the amygdala, bed nucleus of the stria terminalis, basal forebrain, thalamus, and cerebral cortex (Alden et al., 1994; Bernard et al., 1993; Bernard & Besson, 1990; de Lacalle & Saper, 2000; Karimnamazi & Travers, 1998; Knyihar‐Csillik et al., 1999; Yamamoto et al., 1992; Yamamoto et al., 1994; Yasui et al., 1989) led to the discovery that *Calca*‐expressing neurons are important for malaise, anorexia, and other aversive states (Campos et al., 2017; Carter et al., 2015; Carter et al., 2013; Chen et al., 2018; Kaur et al., 2017; Palmiter, 2018; Saper, 2016).

Several other studies focused on neurons we now identify within the *Atoh1* macropopulation, including those that relay thermal (Geerling et al., 2016; Nakamura & Morrison, 2008, 2010) or noxious (Barik et al., 2021; Bester et al., 1995; Chiang et al., 2020; Deng et al., 2020) sensory information from the spinal cord to the forebrain. Based on their location, we predict that PB neurons relaying itch (Mu et al., 2017) also lie within the *Atoh1* macropopulation. Rostrally, neurons that express *Cck* (Fulwiler & Saper, 1985; Grady et al., 2020; Hermanson et al., 1998; Zaborszky et al., 1984) and *Runx1* (Zagami & Stifani, 2010) transmit noxious information from the spinal cord to the hypothalamus (Bester et al., 1995; Hermanson, Larhammar, et al., 1998). Fewer than half of these neurons contain FoxP2 (Garfield et al., 2014; Grady et al., 2020), but the entire *Runx1* population fails to develop in *Atoh1*‐knockout mice (Zagami & Stifani, 2010).

A functional role remains to be determined for many PB neurons, including many of the neurons identified here as expressing *Grp*. Also, we do not yet know the ontological relationships of PB neurons expressing *Oxtr1* (water intake), *Htr2c* (food, water, and salt intake), or *Oprm1* (opioid‐withdrawal; opioid‐induced respiratory depression) relative to our developmental‐genetic framework. While investigating these and other genetic markers (unpublished), we expect that transcriptomic analysis will identify additional, functionally distinct subpopulations within each PB macropopulation.

An interesting question is whether *Lmx1* and *Atoh1* derivation confer separate functional themes. For example, a broadly interoceptive theme may befit the *Lmx1* macropopulation. Its neurons overlap axon terminal fields that deliver viscerosensory information from the nucleus of the solitary tract (Geerling & Loewy, 2006b; Herbert et al., 1990; Rinaman, 2010) and an oral‐sensory subregion of the spinal trigeminal nucleus (Dallel et al., 2004). We showed here that the *Lmx1* macropopulation includes *Satb2* gustatory‐relay neurons, as well as *Calca* neurons that activate in response to visceral stimuli, and KF neurons, which receive chemosensory and other viscerosensory input and exert visceral motor effects on airway tone, breathing, and sympathetic function (Chamberlin & Saper, 1992, 1994; Dutschmann et al., 2021; Dutschmann & Herbert, 2006; Yokota et al., 2015).

In contrast, a broadly exteroceptive theme may befit the *Atoh1* macropopulation. Its neurons overlap axon terminal fields that deliver input from the spinal cord and spinal trigeminal nucleus (Roome et al. 2020; Bernard et al., 1995; Blomqvist et al., 1989; Cechetto et al., 1985; Feil & Herbert, 1995; Kitamura et al., 1993; Panneton & Burton, 1985), and it includes subpopulations that relay pain, temperature, and likely also itch information to the forebrain (Barik et al., 2021; Bester et al., 1995; Bourgeais et al., 2001; Deng et al., 2020; Geerling et al., 2016; Mu et al., 2017; Nakamura & Morrison, 2008, 2010; Norris et al., 2021).

However, there may be exceptions to these broad functional themes. Caudal, *Atoh1*‐derived neurons that express *Pdyn* and *Foxp2* may have appetitive, rather than exteroceptive functions (Gasparini et al., 2021; Kim et al., 2020; Lee et al., 2019). And while we do not yet know the connectivity and function of the *Atoh1*‐derived, *Grp*‐expressing neurons that surround the caudal “head” of the superior cerebellar peduncle, this part of the PB may receive primarily interoceptive input (Feil & Herbert, 1995; Herbert et al., 1990). Also, previous investigators highlighted neurons in the lateral PB that receive pain‐related inputs from both the viscera and the body surface and send output to both the amygdala and hypothalamus (Bernard & Besson, 1990; Bernard et al., 1994; Bourgeais et al., 2003; Chiang et al., 2020; Gauriau & Bernard, 2002). While our neuron‐type‐specific axonal tracing revealed a sharp, developmental distinction between PB neurons that send output to the amygdala (*Lmx1*) versus hypothalamus (*Atoh1*), additional work is needed to test whether interoceptive and exteroceptive input connections to this region segregate similarly. Intersectional genetic and viral targeting methods will help address this question to advance our understanding of the neural pathways transmitting specific channels of interoceptive and exteroceptive information to the brain. This information is necessary for understanding pain, alimentary function, and several other homeostatic functions.

### 4.4 Novel insights into the Kölliker–Fuse nucleus

Our most unexpected finding was that intrinsic, molecular features highlight the KF as a diverse blend of overlapping populations. Having identified the colocalization of Lmx1b and FoxP2 here in rats (Miller et al., 2012) and having distinguished these rostral, glutamatergic neurons from GABAergic neurons in the “caudal KF” (Geerling et al., 2017), we expected to find a homogenous KF population. Instead, without imposing any cytoarchitectonic boundaries or other constraints, molecular markers highlighted this region as a focally diverse cluster of intermingled neurons. Specifically, immunolabeling Lmx1b, Phox2b, and FoxP2 revealed that the KF contains at least four different populations of glutamatergic neurons (Figure 10a, d–g), separate from the “caudal KF” population of GABAergic, *Foxp2*‐expressing neurons. None of these populations were identified using cytoarchitectural criteria.

Also, while previous reports suggested that KF neurons are *Atoh1*‐derived (Gray, 2008; van der Heijden & Zoghbi, 2018), we found no more than sparse *Atoh1* reporter expression ventrolateral to the PB. Instead, most neurons in the KF region express *Lmx1b* (alone, or with *Foxp2*). This *Lmx1b*‐expressing majority intermingles with a substantial minority expressing *Phox2b* (alone, or with *Lmx1b*). That is, KF neurons express either *Lmx1b* or *Phox2b*, both of which are absent from *Atoh1*‐derived neurons. This information, combined with our discovery that many KF neurons derive from *Lmx1a*‐expressing precursors, challenges previous claims that KF neurons derive from *Atoh1*‐expressing precursors in the rhombic lip (Gray, 2008; van der Heijden & Zoghbi, 2018). These claims may have resulted from mislocalizing the KF in mice, which skews rostrally relative to rats (Geerling et al., 2017; Yokota et al., 2015).

Discovering that “the” KF encompasses several populations opens opportunities to test which of these populations are responsible for specific respiratory, orofacial, and autonomic activities associated with this region (Chamberlin & Saper, 1992, 1994; Dutschmann & Dick, 2012; Stanek et al., 2016; Varga et al., 2020). Specifically, intersectional genetic targeting methods should help distinguish the functions of KF neurons that express *Lmx1b* (with and without *Foxp2*) or *Phox2b* (with and without *Lmx1b*).

*Foxp*2‐expressing neurons within the *Lmx1* macropopulation are unique to the KF and caudally contiguous “lateral crescent” (Figure 19), so these neurons probably mediate a function exclusive to the KF region. In contrast, *Phox2b*‐expressing KF neurons are contiguous with many similar neurons extending back through the supratrigeminal nucleus and hindbrain reticular formation. It is not yet clear whether the functional role of *Phox2b*‐expressing KF neurons diverges from that of *Phox2b*‐expressing supratrigeminal neurons or the contiguous “dA3” population of *Phox2b*‐expressing interneurons in the hindbrain reticular formation (Gray, 2013; Hernandez‐Miranda et al., 2017; Kang et al., 2007). Many supratrigeminal neurons send output to cranial motor neurons that pattern movements like chewing, licking, and swallowing (Dempsey et al., 2021; Takatoh et al., 2021; Travers & Norgren, 1983), and in mice, the *Phox2b* distribution overlaps the locations of neurons labeled by viral retrograde tracing from motor neurons that control orofacial movements (Takatoh et al., 2021). In contrast, very few *Phox2b*‐expressing neurons project axons to autonomic and respiratory premotor neurons in the ventrolateral medulla in rats (Kang et al., 2007). These observations suggest that *Phox2b*‐expressing KF neurons have cephalic premotor functions similar to premotor neurons in the supratrigeminal region and reticular formation and distinct from the respiratory activities typically associated with the KF.

A more distinguishing characteristic of KF neurons is their ability to disrupt the respiratory rhythm (Chamberlin & Saper, 1994; Dutschmann & Herbert, 1996, 2006; Lumsden, 1923; Marckwald, 1887). The extensive overlap of *Phox2b*‐expressing neurons here suggests that these (putatively orofacial premotor) neurons work in concert with (putatively separate respiratory premotor) neurons that coordinate breathing with speech or swallowing, both of which require precisely timed perturbations of the respiratory cycle. If the *Foxp2* subset of *Lmx1b*‐expressing KF neurons coordinates lingual and orofacial movements (articulation) or laryngeal movements (phonation) with respiration, then their developmental dysfunction could underlie the orofacial apraxia and speech dyspraxia caused by hemizygous inactivating mutations of *FOXP2* (Fisher et al., 1998; Lai et al., 2001; Shriberg et al., 2006; Stanic et al., 2018).

Also relevant to human health is the role of this region in opioid‐induced respiratory depression (Prkic et al., 2012; Varga et al., 2020). Slice recordings identified opioid‐sensitive neurons in or near the KF (Levitt et al., 2015; Levitt & Williams, 2018; Saunders & Levitt, 2020), yet expression of the mu opioid receptor (*Oprm1*) is prominent in *Calca*‐expressing and intermingled neurons located dorsal and caudal to the KF (Chamberlin et al., 1999; Huang et al., 2021). Intersectional genetic targeting methods should allow investigators to determine whether the *Lmx1b* neurons expressing *Foxp2* (KF) or *Calca* (PB) contribute to opioid‐induced respiratory depression.

## 5 CONCLUSION

Developmental‐genetic markers identify the PB as a blend of two, mutually exclusive macropopulations. These two macropopulations, defined by *Lmx1b* and *Atoh1*, communicate with separate neural circuits. We also found that *Lmx1b* (with and without *Foxp2*) and *Phox2b* (with and without *Lmx1b*) identify neuronal subpopulations in the KF. This new, developmental‐genetic framework will help organize future transcriptomic and experimental work involving PB and KF neurons, and using a molecular ontology to identify and compare neurons across species may accelerate the translation of PB‐related discoveries from experimental animals to human patients.

## AUTHOR CONTRIBUTION

JG planned experiments and supervised the project. JG and RM performed histologic staining and confocal microscopy in rat brain tissue. CM performed confocal microscopy and image processing in rat brain tissue. LP, JL, and II bred and perfused Cre‐reporter mice. LP, RM, and JL performed stereotaxic injections. LP, GI, SK, FG, JL, RM, and JG performed histologic staining and microscopy. BF, II, and VC contributed mice (BF), shared mouse brainstem tissue for Cre fate‐mapping (II and VC), and provided critical guidance and feedback regarding brainstem development. RM and JG plotted neurons in rats. SK and YD plotted neurons in mice. SK and JG analyzed data. SK, JG, and DH drafted and edited all figures. SK and JG wrote the manuscript. All authors reviewed and discussed the results and contributed critical feedback and edits that were incorporated into the final manuscript.

### PEER REVIEW

The peer review history for this article is available at https://publons.com/publon/10.1002/cne.25307

## Data Availability

The data that support the findings of this study are available from the corresponding author upon reasonable request.
